# Experimental evidence that dietary antioxidants induce an antiviral transcriptomic signature in a wild seabird

**DOI:** 10.1242/bio.062786

**Published:** 2026-10-29

**Authors:** Manrico Sebastiano, Marcel Eens, Olivier Chastel, Louis Maigné, David Costantini

**Affiliations:** ^1^ Behavioural Ecology and Ecophysiology Group, Department of Biology, University of Antwerp, Universiteitsplein 1, 2610 Wilrijk, Belgium; ^2^ Centre d'Etudes Biologiques de Chizé (CEBC), UMR7372-CNRS/La Rochelle Université, Villiers-en-Boise F-79360, France; ^3^ Groupe d'Étude et de Protection des Oiseaux en Guyane (GÉPOG), 431 route d'Attila Cabassou, Remire-Montjoly, 97354 French Guiana, France; ^4^ Department of Ecological and Biological Sciences, University of Tuscia, 01100 Viterbo, Italy; ^5^ Unité Physiologie Moléculaire et Adaptation (PhyMA), Muséum National d'Histoire Naturelle, CNRS, 57 Rue Cuvier, CP32, 75005 Paris, France

**Keywords:** Conservation physiology, Gene expression, Wild animals, Seabirds, Immunity

## Abstract

Infectious disease emergence in natural systems is driven by complex host–pathogen–environment interactions. Nutritional factors, particularly dietary antioxidants, can modulate physiological states by enhancing immune function and mitigating oxidative damage, but the underlying molecular mechanisms in wildlife remain poorly understood. To bridge this gap, we conducted a transcriptomic analysis of blood samples from both healthy and clinically affected magnificent frigatebirds *Fregata magnificens* chicks treated with either resveratrol (a plant-derived polyphenol with known antiviral and antioxidant properties) or a placebo. Resveratrol supplementation induced significant gene expression changes, particularly in diseased individuals, where we identified the upregulation of ten key genes in treated sick chicks, including the interferon-stimulated genes (*IFI6*, *OASL2*) and immune regulators (*SOCS1*, *PLAC8*); two additional transcripts were unannotated. Furthermore, resveratrol treatment was associated with increased expression of *DDX60*, a potent antiviral factor, and *CYGB1*, which supports cytoprotection. In contrast, healthy chicks showed fewer differentially expressed genes, primarily associated with metabolism and cellular organization. Collectively, our results reveal a transcriptional signature consistent with dietary antioxidants such as resveratrol acting as active molecular modulators of host defense, rather than simple passive antioxidants, although direct evidence of enhanced antiviral protection (e.g. reduced viral load) remains to be established. This study highlights the utility of transcriptomic approaches to link nutritional ecology with disease dynamics, offering a framework for understanding how dietary quality shapes host defense strategies and population resilience under increasing pathogen pressure.

## Teaser

**Summary:** Transcriptomic analysis reveals that resveratrol activates key immune genes in diseased frigatebird chicks, highlighting the role of nutrition in regulating wildlife molecular defenses.

## INTRODUCTION

The emergence of infectious diseases in natural systems is governed by the dynamic interaction among hosts, pathogens, and the environment, with consequences that scale from individual physiology to long-term population viability (Engering et al., 2013; Vicente-Santos et al., 2023). A fundamental distinction in how organisms navigate these interactions lies in the dual strategies of host resistance and host tolerance (Medzhitov et al., 2012). While resistance refers to the host's ability to reduce or eliminate pathogen burden via immune-mediated clearance, tolerance represents the capacity to limit the physiological damage and fitness costs caused by a given pathogen load (Medzhitov et al., 2012; Singh and Best, 2021). Recent outbreaks of avian influenza and other viral pathogens have caused mass mortality events in diverse seabird populations, highlighting how global connectivity, habitat alteration, and climate change can intensify disease dynamics in wild populations (Baker et al., 2022; Couty et al., 2026; Jaeger et al., 2018). Within this host–pathogen framework, nutritional ecology provides a key lens to understand variation in infection outcomes, as resource acquisition and allocation directly shape immune function and life-history trade-offs. Seabirds are vulnerable to recurrent epizootics as their dense colonial aggregations facilitate rapid pathogen dissemination (McCoy et al., 2016), representing a powerful model system. In several tropical seabird colonies in South America, viruses from the Herpesviridae family have been detected in several seabird species (de Thoisy et al., 2009; Ewbank et al., 2023; Sacristán et al., 2024; Sebastiano et al., 2020), but the epidemiology of these infectious agents has not been fully investigated.

Variation in host nutritional state is increasingly recognized as a key ecological driver of infection outcomes, shaping both resistance (pathogen control) and tolerance (damage limitation) mechanisms (Ayres and Schneider, 2012; Råberg et al., 2009). Within the broader ecological context, because both strategies are energetically and nutritionally demanding, the availability of dietary resources is a critical determinant of whether an individual can successfully suppress a pathogen or simply survive its presence (Munteanu and Schwartz, 2022). Nutrition can influence both the susceptibility to and recovery from infection (Kogut, 2009), modulating immunity at multiple levels. A key link between nutrition and immune response involves antioxidants, which limit oxidative damage arising from normal metabolism and immune activation (Costantini, 2014). In birds, meta-analytic evidence indicates that immune activation can generate reactive oxygen species as part of pathogen elimination in diverse bird species, suggesting that antioxidant defenses are of fundamental importance to mitigate the cost of immune responses (Costantini, 2022). For instance, white-browed sparrow weavers, *Plocepasser mahali*, with a well-balanced oxidative status, showed smaller swelling when immune-challenged, highlighting how antioxidant availability can modulate immune expression (Cram et al., 2015). These findings point to a key eco-immunological trade-off: investment in immune defense can increase oxidative damage, and dietary antioxidants may buffer this cost, thereby altering host–pathogen dynamics by sustaining stronger immune responses.

Therefore, not only are dietary antioxidants passive protectors against oxidative stress, but they may also actively modulate immune pathways and infection outcomes. While their therapeutic potential is well established in human clinical trials and mammalian models, where they serve as potent regulators of systemic inflammation (Malaguarnera, 2019), plant-derived polyphenols like resveratrol have also been explored for their immunomodulatory and stress-buffering roles in avian systems. Across vertebrate taxa, resveratrol acts as a signaling molecule that can shift the balance between pro- and anti-inflammatory cytokines, a mechanism that appears highly conserved. In poultry, dietary supplementation of resveratrol increases meat quality (Zhang et al., 2017), reduces inflammatory responses following stress (He et al., 2019), and improves egg production (Zhang et al., 2026). Importantly, beyond its antioxidant capacity, resveratrol exhibits antiviral activity against a range of DNA and RNA viruses (Abba et al., 2015), including members of the Herpesviridae. For instance, resveratrol treatment substantially reduced virus load in several vertebrate species in both *in vivo* and *in vitro* studies (Sebastiano et al., 2016; Zhao et al., 2018). These dual properties – modulation of oxidative balance and direct antiviral effects (Abba et al., 2015; Tamaki et al., 2014) – make resveratrol a particularly relevant candidate for understanding how diet mediates disease outcomes in wild populations exposed to multiple stressors.

In magnificent frigatebird *Fregata magnificens* chicks at Grand Connétable Island, a natural reserve in French Guiana, severe disease outbreaks and associated mortality events have persisted for several years. In chicks, clinical signs of the disease include crusted skin lesions, hyperkeratosis, and massive mortality episodes in up to 90% of the chicks on a yearly basis, which collectively undermine the breeding success and long-term population viability of this population (de Thoisy et al., 2009; Sebastiano et al., 2018). Such extreme mortality highlights how pathogen pressure can become a dominant selective, potentially interacting with nutritional constraints to shape survival trajectories in this colonial species. In 2016, we therefore performed a resveratrol supplementation study in chicks to investigate whether this powerful antioxidant could reduce oxidative stress and improve the short-term progression of the disease and survival perspectives of supplemented birds (Sebastiano et al., 2017a). We found that resveratrol supplementation increased circulating antioxidant defenses and plasma haptoglobin levels (Sebastiano et al., 2018) and reduced the generation of lipid oxidative damage (Sebastiano et al., 2018). Although these results demonstrate that nutritional interventions can alter physiological responses in the wild, the molecular mechanisms linking antioxidant intake to immune function and disease outcomes remain unresolved.

To address this gap, we investigated the molecular pathways modulated by resveratrol supplementation using transcriptomic analyses. We analyzed blood samples from healthy and clinically affected chicks that received either a resveratrol supplementation or a placebo. Transcriptomic profiling provides an unbiased, genome-wide window into the molecular mechanisms underlying ecologically relevant phenotypes, linking nutritional inputs to measurable shifts in immune strategy (Eskew et al., 2021). Based on current eco-immunological theory regarding the dual role of antioxidants in mediating resistance and tolerance (Ayres and Schneider, 2012; Medzhitov et al., 2012), we formulated the following hypotheses and predictions. First, we hypothesized that dietary antioxidant supplementation enhances host resistance to infection by upregulating innate antiviral immune pathways (Hypothesis 1). Specifically, we predicted that resveratrol-supplemented chicks would exhibit increased expression of interferon-stimulated genes and genes involved in early innate immune signaling and cytokine regulation. Second, we hypothesized that dietary antioxidants enhance host tolerance by reducing oxidative stress and limiting immunopathology associated with infection (Hypothesis 2). We further predicted that these transcriptomic responses would be more pronounced in clinically affected chicks than in apparently healthy chicks, because active disease may increase both immune activation and oxidative stress, thereby increasing the scope for resveratrol to modulate these pathways. Because clinical status itself is expected to influence gene expression, comparisons between health-status groups were interpreted separately from treatment effects. Finally, we evaluated how transcriptomic approaches in wild systems can bridge the gap between nutritional ecology, immune function, and disease dynamics, providing a mechanistic framework for understanding host–environment interactions in the face of emerging infectious diseases.

## RESULTS

### Differentially expressed genes (DEGs)

When comparing clinically affected chicks that received resveratrol supplementation (*n*=6) with those that received a placebo (*n*=6), we identified a total of ten DEGs, of which two are unannotated novel transcripts, all of which were significantly upregulated in resveratrol-treated individuals relative to controls (Table 1, Fig. 1). After two novel, unannotated transcripts (FREMAG_R14896 and FREMAG_R14315, both of which lack known orthologs in the reference annotation and were therefore not considered for biological interpretation; Table 1) were excluded, the IFI6 (Interferon alpha Inducible Protein 6) gene showed the greatest increase in expression as compared to chicks that did not receive resveratrol. PLAC8 (Placenta-specific 8, 84.5-fold increase), CAMKK2 (Calcium/calmodulin-dependent protein kinase kinase 2, 15.2-fold increase), OASL2 (2′-5′-oligoadenylate synthetase-like 2, 15.9-fold increase), BAIAP2L2 (BAR/IMD Domain-Containing Adapter Protein 2-Like 2, 9.3-fold increase), SOCS1_1 (Suppressor of Cytokine Signaling 1, 7.9-fold increase), CEP70_0 (Centrosomal Protein 70, 4.6-fold increase), and BTN1A1_0 (Butyrophilin 1A1, 2.7-fold increase) were also highly expressed in resveratrol-supplemented birds compared to control birds. Box plots of each individual DEG can be found in Fig. S1.

**Fig. 1.**
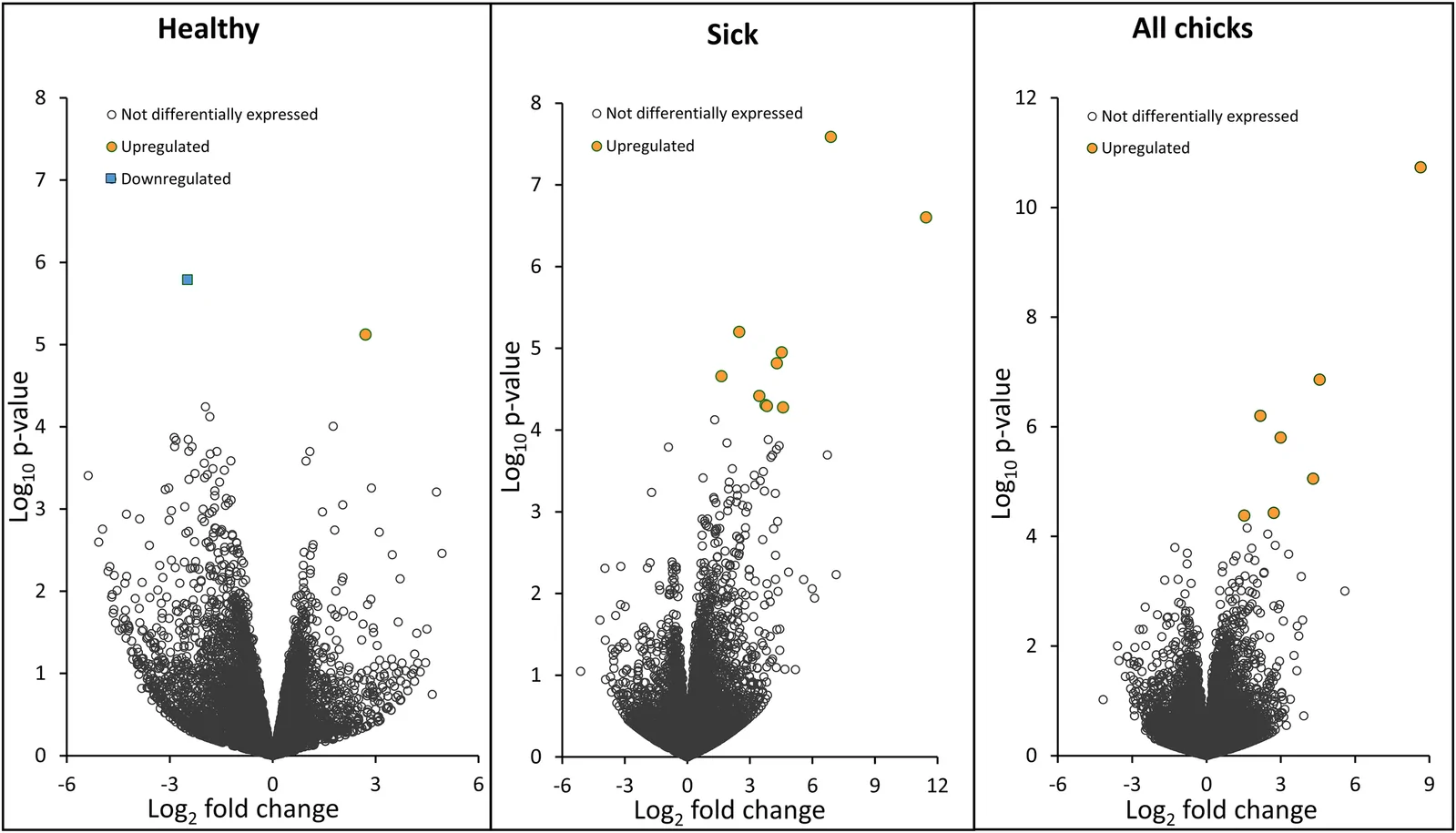
**Volcano plots of gene expression levels between birds that were supplemented with resveratrol pills and those that received a placebo.** DEGs (genes with an adjusted *P*-value <0.05 and a log2 fold change above 1 or below −1) between magnificent frigatebird chicks from French Guiana in healthy, sick, and all chicks combined, are coloured according to the figure legend.

**Table 1.** DEGs with the highest log2 fold change and the lowest adjusted *P*-value between sick birds (magnificent frigatebird chicks from French Guiana) that were treated with resveratrol pills (*n*=6) and those that received a placebo (*n*=6); between healthy chicks that received resveratrol (*n*=3) and those that received a placebo (*n*=5); and between chicks that received resveratrol (*n*=9) and those that received a placebo (*n*=11) when pooled – i.e. without considering their health status

Gene ID	Resveratrol	Placebo	Fold change (shrunk)	Log2 fold change (shrunk)	*P*-adj	Gene name	Description
**Sick chicks**							
Gene-FREMAG_R01373	24,689	9	1924	10.9	<0.001	IFI6	Immunity
Gene-FREMAG_R04600	2996	26	84.5	6.4	<0.001	PLAC8	Cell proliferation, immunity
Gene-FREMAG_R10109	251	45	4.6	2.2	0.018	CEP70_0	Cytoskeleton assembly
Gene-FREMAG_R03004	8193	356	15.9	4.0	0.024	OASL2	Innate immune response
Gene-FREMAG_R14896	208	11	13.6	3.8	0.026	FREMAG_R14896	NA
Gene-FREMAG_R01377	2419	783	2.7	1.4	0.032	BTN1A1_0	Immunity
Gene-FREMAG_R05260	497	46	7.9	3.0	0.045	SOCS1_1	Cytokine signaling, immunity
Gene-FREMAG_R01491	194	14	9.3	3.2	0.045	BAIAP2L2	Membrane structures
Gene-FREMAG_R14315	101	7	9.7	3.3	0.045	FREMAG_R14315	NA
Gene-FREMAG_R08715	128	5	15.2	3.9	0.045	CAMKK2	Calcium regulation
**Healthy chicks**							
Gene-FREMAG_R09204	150	838	−4.7	−2.2	0.009	SPTBN4	Structural organization
Gene-FREMAG_R08275	94	14	5.3	2.4	0.029	ENTPD3	Signaling regulation
**All chicks**							
Gene-FREMAG_R01373	17,001	11	1267	10.3	<0.001	IFI6	Immunity
Gene-FREMAG_R04600	2093	27	64.3	6.0	<0.001	PLAC8	Cell proliferation, immunity
Gene-FREMAG_R10109	196	43	4	2	0.002	CEP70_0	Cytoskeleton assembly
Gene-FREMAG_R05260	364	46	6.5	2.7	0.003	SOCS1_1	Cytokine signaling, immunity
Gene-FREMAG_R14896	145	7	14.1	3.8	0.013	FREMAG_R14896	NA
Gene-FREMAG_R09551	1783	272	5.1	2.4	0.043	DDX60	Antiviral factor
Gene-FREMAG_R03150	59	20	2.5	1.3	0.043	CYGB1	Cytoprotection

Genes are sorted based on their adjusted *P*-values. Data are expressed as normalized read counts.

When comparing chicks without visible clinical symptoms that received resveratrol supplementation (*n*=3) with those that received a placebo (*n*=5), we found a total of two DEGs (Table 1, Fig. 1). The ENTPD3 (Ectonucleoside Triphosphate Diphosphohydrolase 3) was upregulated by 5.3-fold in healthy chicks that received resveratrol, while the SPTBN4 (Spectrin Beta, Non-Erythrocytic 4) was downregulated by 4.7-fold in chicks that received resveratrol.

Finally, when comparing all chicks that received resveratrol (*n*=9) with those that received a placebo (*n*=11) – i.e. irrespective of their health status – we found seven DEGs, all of which were upregulated in chicks that received resveratrol compared to chicks that received a placebo (Table 1, Fig. 1). One novel gene transcript that did not match a sequence in available databases was excluded (Table 1). Of the remaining genes, four upregulated genes were similar to those found in sick chicks (IFI6, PLAC8, SOCS1_1, CEP70_0), while there were also two genes, namely, DDX60 (DExD/H-box helicase 60) and CYGB1 (Cytoglobin 1), that were both upregulated by 5.1- and 2.5-fold, respectively, in treated chicks.

### GO enrichment analysis of DEGs

To further explore the biological functions and the classification of DEGs, we used enrichment analysis to identify specific classes of genes that have been affected by resveratrol supplementation. GO classification showed no terms in any of the BP, CC, and MF categories when sick and healthy chicks were grouped separately (Table 2; Figs S2 and S3, Appendix A). However, when chicks were pooled irrespective of their health status, we found that the GO term ‘extracellular region’ from the CC category was significantly enriched by a mix of upregulated and downregulated DEGs (Fig. 2; Fig. S4, Appendix A), while six GO terms from the MF category (hormone activity, transferase activity, MF regulators, NAD+ ADP-ribosyltransferase activity, receptor regulator activity, and receptor ligand activity) were also significantly enriched by a mix of upregulated and downregulated DEGs (Table 2, Fig. 2; Fig. S4, Appendix A).

**Fig. 2.**
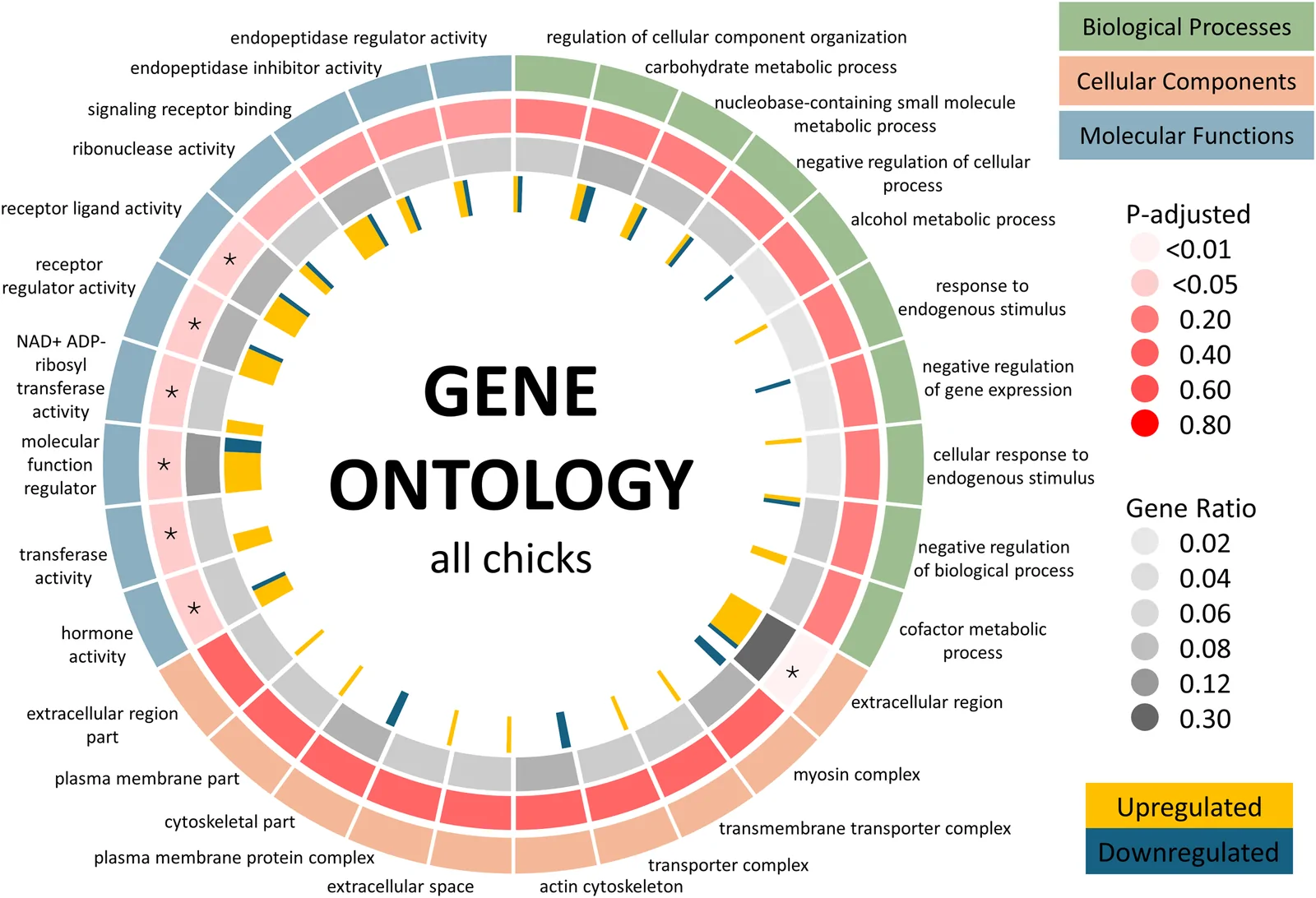
**Circular map of the top ten GO term classifications of DEGs for each GO term category between birds that were supplemented with resveratrol pills and those that received a placebo in magnificent frigatebird chicks from French Guiana.** The first (outer) circle: different colors represent different GO term categories: BPs, CCs, and MFs. The second circle: adjusted *P*-values colored by their significance level according to the legend (asterisks indicate significant GO terms). The third circle: gene ratio (the number of DEGs enriched in each pathway divided by the total number of background genes enriched in this pathway) colored by their significance level according to the legend. The fourth (inner) circle: the number of upregulated (in yellow) and downregulated genes (in blue) in each GO term.

**Table 2.** GO classification of DEGs in magnificent frigatebird chicks from French Guiana facing a viral disease, upregulated or downregulated in chicks treated with resveratrol (*n*=9) versus those receiving a placebo (*n*=11), independently from their health status

Category	GO ID	Description	Gene ratio	Up	Down	*P*-adj
CC	GO:0005576	Extracellular region	10/34	9	1	<0.001
MF	GO:0005179	Hormone activity	5/117	4	1	0.02
MF	GO:0016763	Transferase activity, transferring pentosyl groups	4/117	4	0	0.02
MF	GO:0098772	MF regulator	13/117	9	4	0.04
MF	GO:0003950	NAD+ ADP-ribosyltransferase activity	3/117	3	0	0.04
MF	GO:0030545	Receptor regulator activity	7/117	6	1	<0.05
MF	GO:0048018	Receptor ligand activity	7/117	6	1	<0.05

Gene ratio refers to the percentage of total DEGs in the given GO term.

### Kyoto Encyclopedia of Genes and Genomes (KEGG) enrichment analysis of DEGs

We performed KEGG enrichment analysis to further understand which pathways were significantly enriched by DEGs between the studied phenotypes and experimental groups. When sick chicks that received a resveratrol supplementation were compared with those that received a placebo, none of the KEGG pathways were significantly enriched (Fig. S5, Appendix A). When healthy chicks that received a resveratrol supplementation were compared with those that received a placebo, two KEGG pathways were significantly enriched and were mainly involved in the cytoskeleton of muscle cells and focal adhesion, both of which are structural components of cells (Table 3, Fig. 3; Fig. S6, Appendix A). Finally, when all chicks that received either resveratrol supplementation or a placebo were pooled without considering their health status, none of the KEGG pathways were significantly enriched (Fig. S7, Appendix A).

**Fig. 3.**
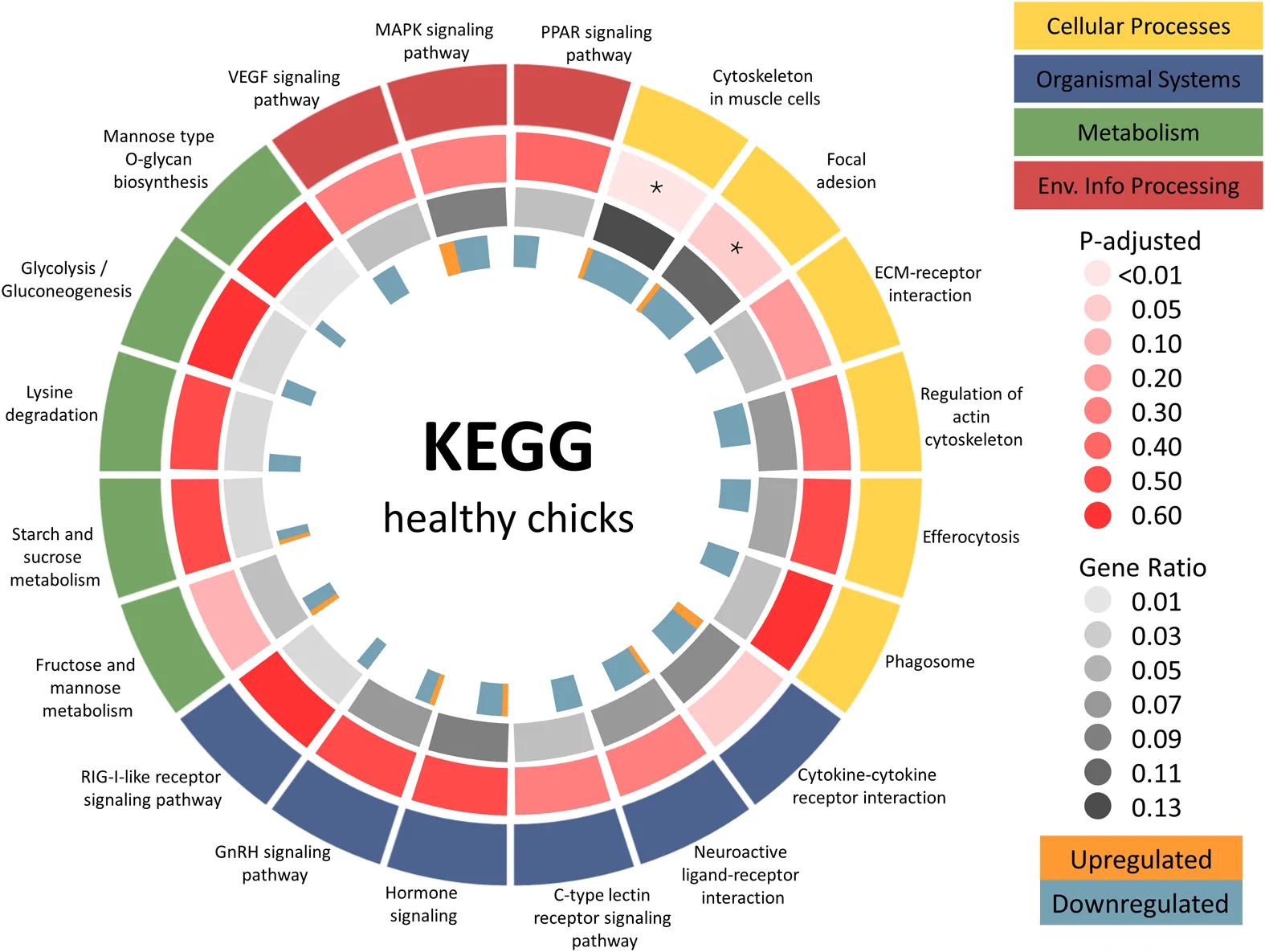
**Circular map of the top 20 KEGG pathways enriched by DEGs between healthy birds that were supplemented with resveratrol pills and those that received a placebo in magnificent frigatebird chicks from French Guiana.** The first (outer) circle: different colors represent different KEGG A classes: cellular processes, organismal systems, metabolisms, and environmental information processing. The second circle: adjusted *P*-values colored by their significance level according to the legend (asterisks indicate significant KEGG pathways). The third circle: gene ratio (the number of DEGs enriched in each pathway divided by the total number of background genes enriched in this pathway) colored by their significance level according to the legend. The fourth (inner) circle: the number of upregulated (in orange) and downregulated genes (in blue) in each KEGG pathway.

**Table 3.** KEGG classification of DEGs downregulated or upregulated in healthy chicks that received resveratrol supplementation (*n*=3) or a placebo (*n*=5)

KEGG ID	Description	Gene ratio	Up	Down	*P*-adj
ccw04820	Cytoskeleton of muscle cells	17/138	1	16	0.002
ccw04510	Focal adhesion	14/138	1	13	<0.05

Gene ratio refers to the percentage of total DEGs over the total genes in a given pathway.

## DISCUSSION

Our results provide, for the first time, experimental evidence in a natural system that dietary antioxidants can modulate immune gene expression in a wild host–pathogen interaction, with functional implications for both resistance and tolerance strategies. Resveratrol supplementation induced marked molecular changes in magnificent frigatebird chicks, particularly in individuals displaying clinical signs of a viral disease. Although the number of DEGs was limited, their functional identity reveals coordinated shifts in antiviral defense and cellular stress responses, consistent with targeted rather than systemic transcriptional reprogramming.

The strong upregulation of interferon-stimulated and antiviral genes in clinically affected chicks receiving resveratrol, particularly IFI6, OASL2, and SOCS1, is consistent with activation of antiviral signaling pathways central to early immune defense, although in the absence of a direct measure of viral load in these birds, we cannot confirm whether this transcriptional response translated into reduced pathogen burden. IFI6 is a well-known interferon-inducible gene involved in limiting viral replication and promoting infected cell survival during antiviral responses (Villamayor et al., 2023). Its strong upregulation in resveratrol-treated sick chicks indicates activation of interferon-mediated antiviral pathways despite short-term supplementation, highlighting the responsiveness of these pathways to nutritional inputs. Similarly, OASL2 and SOCS1 genes are central components of innate immunity. Like IFI6, OASL2 is an interferon-inducible gene that boosts immune defenses by suppressing viral replication and by activating retinoic-acid-inducible gene signaling (Zhu et al., 2014), the first line of defense against viruses (Rehwinkel and Gack, 2020; Thoresen et al., 2021). From an ecological perspective, such modulation of innate immunity may alter infection dynamics at the individual level, potentially reducing viral replication, and is consistent with improved regulation of immune-mediated damage, pointing toward enhanced tolerance. SOCS1, which is fundamental for the negative regulation of inflammatory cytokine and chemokine production; activation of microglia, macrophages and astrocytes; immune cell infiltration; and autoimmunity (Baker et al., 2009), was also highly expressed in resveratrol-supplemented chicks, suggesting tighter control of inflammatory responses. These patterns support the dual action of dietary antioxidants – i.e. enhancing antiviral defense (resistance) while limiting immunopathology (tolerance) – which aligns with the eco-immunological theory.

Two additional upregulated genes, PLAC8 and BTN1A1, are nowadays recognized as fundamental in immune-regulatory functions (Wang et al., 2020; Zhang et al., 2024). This supports the idea that resveratrol, and possibly other polyphenols, acts as an active modulator of immune signaling rather than a passive antioxidant. These findings are consistent with experimental evidence showing that resveratrol reduces viral load and replication in vertebrate hosts, including herpesvirus models (Abba et al., 2015; Sebastiano et al., 2016). In sick chicks, we have also found that resveratrol stimulated the expression of genes involved in cytoskeleton assembly (i.e. the CEP70 gene; Shi et al., 2011), membrane structures (i.e. BAIAP2L2; Yan et al., 2022), and a calcium-dependent protein kinase involved in several cellular processes (i.e. CAMKK2; Sabbir et al., 2021). A recent review showed that alterations in cytoskeletal architecture influence host–pathogen interactions (Taylor et al., 2011), as many viruses exploit the host cytoskeleton for entry, replication, and diffusion (De Conto et al., 2025; Taylor et al., 2011). Taken together, the upregulation of such genes highlights the complexity of host–pathogen responses, which are therefore not confined to pure antiviral functions and immune signaling but also to structural and cellular resilience molecular mechanisms modulated by the host nutritional status.

When both healthy and sick chicks were pooled, we observed strikingly similar immune responses, likely because clinically affected individuals comprised more than 50% of this group. In addition to those genes that were equally upregulated in sick chicks (i.e. IFI6, PLAC8, CEP70, and SOCS1), we found that resveratrol supplementation upregulated the expression of DDX60, a powerful antiviral factor (Miyashita et al., 2011), and of CYGB1, a gene coding for a protein that functions primarily in cytoprotection against oxidative stress (Yoshizato et al., 2016). The upregulation of such genes provides a molecular signature consistent with enhanced host tolerance, as these responses may help limit the physiological damage caused by the pathogen without necessarily reducing its load. This is consistent with our previous findings showing increased oxidative damage in untreated sick chicks (Sebastiano et al., 2018). In that earlier study using the same experimental population, resveratrol-supplemented chicks showed higher circulating antioxidant defenses and plasma haptoglobin and reduced lipid oxidative damage relative to placebo-treated birds (Sebastiano et al., 2018). The present transcriptomic results extend those physiological findings to the molecular level: the upregulation of CYGB1, a cytoprotective gene induced by oxidative and hypoxic stress, together with the broader antiviral and immunoregulatory signature described above, is consistent with a mechanistic link between antioxidant intake and reduced immune-associated cellular damage. We did not, however, test this link statistically at the individual level (e.g. by relating antioxidant or oxidative-damage values to gene expression for each bird), and we considered such an integrated analysis an important next step rather than a claim supported by the present dataset. Taken together, these results strengthen the view that dietary antioxidants function as regulators of physiological state, linking nutritional condition to immune performance and damage limitation. In contrast, healthy chicks exhibited a much more restricted transcriptional response, with only two DEGs between the resveratrol-supplemented and control birds. The upregulation of ENTPD3, a key enzyme in nucleotide metabolism and in processes like energy metabolism and nucleotide hydrolysis (Saunders et al., 2019), may have significant implications for enhancing systemic energy homeostasis during growth. In contrast, the other DEG between resveratrol-supplemented and control chicks was SPTBN4, which is involved in the organization of the cytoskeletal membrane (Ibrahim et al., 2024). This suggests that in the absence of infection, resveratrol primarily influences baseline metabolic and structural processes rather than activating immune pathways, consistent with the context-dependent effects of nutritional interventions.

At the systems level, GO enrichment analyses further support a role for resveratrol in modulating intercellular communication and systemic signaling. When chicks were analyzed irrespective of health status, enriched GO terms included ‘extracellular region’, ‘hormone activity’, and ‘receptor regulation’. This pattern suggests that resveratrol influences the production and regulation of secreted factors, including cytokines and hormones, which coordinate organism-wide responses to infection. The enrichment of regulatory functions indicates that supplementation may alter how cells perceive and integrate environmental and physiological signals (Eskew et al., 2021). Such effects are particularly relevant in a nutritional ecology context, where diet-mediated changes in signaling pathways can shape life-history trade-offs between growth, immunity, and stress responses. Resveratrol is widely reported to modulate inflammatory signaling by interfering with key transcription factors essential for limiting immunopathology, including nuclear factor kappa B (NF-κB) and mitogen-activated protein kinase pathways (MAPK) (Baur and Sinclair, 2006; Medzhitov et al., 2012; Shakibaei et al., 2009). Finally, the enrichment of the GO term related to hormone activity is also biologically meaningful in the context of seabird chicks experiencing infection and physiological stress. In birds, infection often interacts with endocrine axes that regulate growth, energy allocation, and stress responsiveness, including glucocorticoids (Sapolsky et al., 2000). However, because these patterns emerged only in pooled analyses, future studies with larger sample sizes and longitudinal designs will be necessary to determine whether these transcriptional shifts represent early regulatory responses that precede phenotypic changes in disease progression.

KEGG enrichment analyses in healthy chicks highlighted pathways related to cytoskeletal organization and focal adhesion. While these results should be interpreted cautiously given the small sample size, they raise the possibility that resveratrol may influence cellular architecture and tissue maintenance even in the absence of the disease, as previously observed (Csiszar et al., 2009). As a predominant downregulation of genes was observed (16/17 and 13/14 genes downregulated), this more plausibly reflects reduced structural/cytoskeletal investment, rather than a strengthening of tissue barriers against future pathogen-induced damage (Ayres and Schneider, 2012), as hypothesized.

Finally, we would like to point out that a key limitation of this study is the small number of biological replicates underlying the DESeq2 contrasts, particularly for the healthy-chick comparison (*n*=3 resveratrol versus *n*=5 placebo). Benchmarking the work of Schurch et al. (2016) showed that at three replicates per group, RNA-sequencing differential expression analyses recover only ∼20-40% of true DEGs overall. Under this framework, the lower-fold DEGs identified in this study, such as BTN1A1 (2.7-fold), CYGB1 (2.5-fold), and CEP70 (4.6-fold), fall within a range where both sensitivity and reproducibility are expected to be low at these sample sizes. We therefore interpret these three genes with caution and note that they should be treated as provisional until confirmed in a larger sample. The healthy-chick contrast as a whole, including its associated KEGG enrichment results (cytoskeleton of muscle cells, focal adhesion), sits below the replicate number generally considered sound and should accordingly be regarded as exploratory rather than conclusive. Future studies should (i) consider larger sample sizes, particularly for the healthy-chick comparison, to confirm these lower-magnitude transcriptional responses and (ii) validate DEGs via real-time quantitative polymerase chain reaction (qPCR) to increase confidence in the interpretation of biological responses (Conesa et al., 2016). As in the present study, we did not collect a differential leukocyte count or other measures of blood cell composition; some DEGs, particularly the structural/cytoskeletal terms in healthy chicks, may reflect shifts in circulating cell-type proportions, and such results should be interpreted with caution. In addition, viral load and herpesvirus presence were not quantified in the sequenced birds; elevated expression of interferon-stimulated genes is consistent with but does not on its own demonstrate reduced pathogen burden, and it may also partly reflect among-individual variation in infection stage. This constrains our ability to firmly distinguish resistance from tolerance mechanisms in the resveratrol-supplemented sick chicks. Future work could extend these findings using single-cell transcriptomics on peripheral blood mononuclear cells to identify which specific cell subpopulations drive the observed transcriptional response, potentially revealing additional DEGs masked by the bulk sequencing approach used here. Furthermore, combining transcriptomic data with proteomic validation of the key transcripts identified would confirm their biological relevance at the protein level. Both approaches were beyond the scope and resources of the present study.

### Conclusions

This study demonstrates that nutritional antioxidants can reshape the immune defense in wild hosts, linking dietary quality to molecular pathways relevant to disease outcomes. By influencing both antiviral defense and the regulation of inflammation and inflammatory damage, resveratrol affects key molecular components of both resistance and tolerance, thereby linking nutritional inputs to fundamental eco-evolutionary processes governing disease outcomes. In colonies experiencing high pathogen pressure like ours (up to 90% mortality of chicks in some years; Sebastiano et al., 2018), even modest improvements in disease resistance or tolerance could have significant demographic consequences.

More broadly, our findings position nutritional ecology as a central component of disease ecology, highlighting how variation in resource quality can cascade from molecular pathways to population-level dynamics. Future work combining transcriptomics with direct measures of pathogen load, immune cell profiles, and long-term fitness outcomes will be essential to fully elucidate these relationships in natural contexts.

Finally, our study illustrates how transcriptomic approaches can bridge the gap between ecological and molecular mechanisms, providing a powerful framework to uncover hidden physiological trade-offs (Eskew et al., 2021). By connecting diet, gene expression, and disease outcomes, such approaches offer a mechanistic basis for predicting how environmental change – including shifts in resource availability – will influence host susceptibility and population resilience in the wild, before large-scale demographic changes occur.

## MATERIALS AND METHODS

### Sample collection

We conducted fieldwork in 2016 on Grand Connétable Island, a protected breeding site for seabirds located off the northern Atlantic coast of South America (French Guiana; 4°49′30″N, 51°56′00″W). The island supports approximately 1300 breeding pairs of frigatebirds [Groupe d'Etude et de Protection des Oiseaux en Guyane (GEPOG) field observations]. In this colony, most pairs initiate breeding between late November and early December. As a result, all chicks were of similar developmental stage at the time of capture, with an estimated age of approximately 4 months, as supported by statistical analyses in Sebastiano et al. (2018). Repeated sampling of chicks is easily achievable in this species, as they do not move from their nests until they are a few months old.

We randomly selected 60 chicks from different areas of the island. Of these, 26 individuals showed no visible signs of disease, whereas 34 exhibited clear clinical symptoms. We classified diseased chicks based on the presence of crusted lesions on the head and body and hyperkeratosis around the eyes, following previous work (de Thoisy et al., 2009). We captured all chicks by hand at their nests on 7 June (D1). Within 3 min after capture, we collected 2 ml of blood from the brachial vein using a heparinized syringe and a 25G needle. We then ringed each bird with an aluminum band for individual identification and measured beak–head length to control for variation in chicks' ages. These initial blood samples were used to assess pre-treatment differences among experimental groups.

We initiated the experimental treatment on D2. We administered *trans*-resveratrol (see Table S2) to 12 of 26 healthy nestlings and 18 of 34 diseased nestlings. The remaining individuals (14 healthy and 16 sick chicks) received an empty capsule as a placebo to control for handling effects. We selected resveratrol because of its strong antiviral activity against herpesviruses (Abba et al., 2015; Sebastiano et al., 2016), which could be actively replicating in clinically affected frigatebird chicks (de Thoisy et al., 2009). To facilitate swallowing, we coated all capsules in fish oil (Crafty Catcher, Ipswich, UK). We administered treatments on nine occasions across three periods: D2-D4 (8-10 June), D12-D14 (18-20 June), and D21-D23 (27-29 June).

We ended the experiment on D24, when we collected a second 2 ml blood sample from each individual. We kept blood samples refrigerated in the field and centrifuged them within 2 h to separate plasma and red blood cells. We used plasma for measurements of corticosterone, oxidative stress biomarkers, and inflammatory and immune parameters in another study (Sebastiano et al., 2017b). We stored all samples on dry ice during fieldwork and subsequently transferred them to a −80°C freezer upon arrival at the laboratory. To quantify changes in disease severity, we photographed each bird at a standardized distance and position at both the beginning and end of the experiment, enabling us to score changes in visible clinical signs over time as described by Sebastiano et al. (2018). All procedures were approved by the Ethical Committee of the University of Antwerp and by DEAL Guyane (Direction de l'Environnement, de l'Aménagement et du Logement), and fieldwork was conducted under permit R03-2013-06-01-007 issued by the Préfet de la Région Guyane.

### RNA extraction and sample quality-control

A total of 20 birds had a clear health history (i.e. precise information on the health status before and after the experiment) and were therefore selected for RNA extraction and transcriptomic analyses, of which eight birds belonged to the healthy group (three supplemented with resveratrol and five receiving a placebo) and 12 belonged to the sick group (of which six were supplemented with resveratrol and six received a placebo). Frozen blood samples were shipped to and processed by Novogene GmbH, where RNA was extracted (using an in-house confidential protocol developed by Novogene) and where RNA quality was checked using an Agilent 5400 for quantification, integrity, and purity (Table S1). Messenger RNA was purified from total RNA using poly-T oligo-attached magnetic beads. Library construction was carried out using the NEBNext^®^ Ultra™ RNA Library Prep Kit for Illumina^®^, following manufacturer instructions. After fragmentation, the first-strand cDNA was synthesized using random hexamer primers, followed by the second-strand cDNA synthesis using either deoxyuridine triphosphate (dUTP) for directional library or deoxythymidine triphosphate (dTTP) for nondirectional library. The library was ready after end repair, A-tailing, adapter ligation, size selection, amplification, and purification. The library was checked with Qubit and real-time PCR for quantification and Agilent 2100 Bioanalyzer for size distribution detection. Quantified libraries were pooled and sequenced on Illumina Sequencing PE150 platforms, according to effective library concentration and data amount.

### Bioinformatic analyses

The raw sequencing reads for all 20 samples have been deposited in the National Center for Biotechnology Information (NCBI) Sequence Read Archive under BioProject accession PRJNA1519313. Raw sequencing reads in FASTQ format were quality filtered by removing adapter sequences, poly-N reads, and low-quality reads to obtain clean data. At the same time, Q20 and Q30 quality scores and the guanine–cytosine (GC) content were calculated. All the downstream analyses used only high-quality clean data. Reads were aligned to the *F. magnificens* reference genome assembly ASM1338994v1 (GenBank accession GCA_013389945.1; SOAPdenovo v2.04, ∼40× Illumina HiSeq coverage), generated as part of the Bird 10,000 Genomes (B10K) Project (Feng et al., 2020), using HISAT2 v2.0.5 (Kim et al., 2019). Gene identifiers and symbols follow B10K project's comparative annotation, in which gene models are projected onto each genome via a reference-free whole-genome alignment (Cactus) using conserved synteny with the chicken (*Gallus gallus*) reference annotation (Feng et al., 2020); numeric suffixes (e.g. _0 and _1) indicate multiple loci projected onto the same base gene symbol. The mapped reads of each sample were assembled by StringTie v2.2.3 (Pertea et al., 2015) in a reference-based approach. FeatureCounts v2.0.6 (Liao et al., 2014) was used to count the number of reads mapped to each gene. Differential expression was assessed using DESeq2 (1.42.0) with a simple one-factor design (∼condition), comparing resveratrol-supplemented and placebo-treated chicks separately within sick, healthy, and pooled groups. No additional covariates were modeled. Sex was not included because it was not determined for the sequenced individuals. We ran the model on read counts adjusted through one scaling normalized factor, a method for differential analysis of count data, using shrinkage estimation for dispersions and fold changes to improve stability and interpretability of estimates (Love et al., 2014). We further performed differential expression analysis between sick birds that received a resveratrol pill or those that received a placebo and between healthy chicks that received a resveratrol pill or those that received a placebo. Log2 fold changes were shrunk using the apeglm estimator (Zhu et al., 2019) as implemented in DESeq2's lfcShrink function, and shrunken values are reported throughout in place of raw maximum-likelihood estimates. Note that the pooled contrast (resveratrol-supplemented versus control birds, irrespective of health status) is not statistically independent of the sick-chick contrast, since sick chicks make up >50% of the pooled sample. Independent filtering and outlier exclusion (Cook's distance) were applied separately per contrast. In the sick-chick contrast, of 11,633 genes with nonzero counts, 88 (0.76%) were excluded as count outliers and 2913 (25%) by independent filtering (mean count threshold=4), leaving 8632 genes tested. In the healthy-chick contrast of 11,611 genes, five (0.043%) were excluded as outliers and none by independent filtering (threshold=0); one further gene, *Col4a1*, was excluded from interpretation despite meeting nominal significance and fold-change thresholds, as it showed no detectable expression in most samples (zero counts in all three resveratrol-treated birds and three of five controls) – apeglm shrinkage collapsed its log2 fold change to approximately zero, consistent with this being a numerical artifact rather than a genuine effect. In the pooled contrast, of 12,264 genes, none were excluded as outliers, and 4999 (41%) were excluded by independent filtering (threshold=15), leaving 7265 genes tested. All tests were corrected for multiple comparisons using the Benjamini–Hochberg method.

We set thresholds for differential expression at adjusted *P*-values <0.05 and absolute log2 fold change above 1 (or below −1) – corresponding to at least a twofold difference between groups. We used adjusted *P*-values <0.05 for visual representation in the volcano plot (Fig. 1). We reported novel gene transcripts that did not match a sequence in available databases in tables within Appendix A, but we did not further discuss those genes in relation to the interpretation of the results.

### Enrichment analyses of DEGs

We carried out enrichment analysis to find out which biological functions or pathways are significantly associated with DEGs. GO enrichment analysis of DEGs was implemented using clusterProfiler (4.8.1), in which gene length bias was corrected. GO terms with corrected *P*-values less than 0.05 were considered significantly enriched by DEGs. We used GO to ascribe biological processes (BPs), molecular functions (MFs), and cellular components (CC) of genes in a directed acyclic graph structure. For visual representation of these analyses, GO graphs considered the first ten terms with the lowest *P*-value of each category of genes. We further used KEGG to understand high-level functions and utilities of genes (Kanehisa et al., 2016). We used the clusterProfiler R package to test the statistical enrichment of differentially expressed genes in KEGG pathways. For visual representation, the KEGG graph included the first 20 terms with the lowest *P*-value. When GO and KEGG terms showed an adjusted *P*-value below 0.05, we considered them significantly enriched by DEGs and thus included them in the Results and discussed them in the Discussion section.

### Declaration of generative artificial intelligence (AI) and AI-assisted technologies in the writing process

During the preparation of this work, the first author used ChatGPT-5.6 Luna version only to improve language and readability. After using this tool/service, the authors reviewed and edited the content as needed and take full responsibility for the content of the publication.

## Supplementary Material



10.1242/biolopen.062786_sup1 Supplementary information

Table S2.
