# Supplementary material for: Experimental evidence that dietary antioxidants induce an antiviral transcriptomic signature in a wild seabird
Source: Biol Open. 2026 Oct 29;15(10):bio062786. doi: 10.1242/bio.062786 (PMC13750391; doi:10.1242/bio.062786)
Supplement: Supplementary file 1 [file biolopen-15-062786-s1.pdf]

Table S1. RNA quality control metrics for the 20 samples used in transcriptomic analysis. *Status*: health status of the chick at sampling (Healthy or Sick). *Treatment*: experimental group (Resveratrol or Control/placebo). *Nucleic Acid ID*: Novogene internal sample identifier. *C (ng/μl)*: RNA concentration. *Vol. (μl)*: sample volume submitted for sequencing. *Amount (μg)*: total RNA amount (concentration × volume). *Integrity value (RIN)*: RNA Integrity Number, assessed via Agilent 5400 (Novogene). All samples passed Novogene's standard RNA QC criteria prior to library preparation and sequencing.

No.	Sample Name	Status	Treatment	Nucleic Acid ID	C (ng/ul)	Vol. (ul)	Amount(ug)	Integrity value (RIN)
1	FB_14_H2_RES	Healthy	Resveratrol	EZTR250005929-2A	69.21	37	2.56	4.6
2	FB_16_H2_RES	Healthy	Resveratrol	EZTR250005930-1A	29.02	26	0.75	5.9
3	FB_20_H2_RES	Healthy	Resveratrol	EZTR250005931-1A	16.53	26	0.43	5.1
4	FB_2_H2_C	Healthy	Control	EZTR250005932-1A	22.19	26	0.58	5
5	FB_9_H2_C	Healthy	Control	EZTR250005933-1A	40.63	26	1.06	5
6	FB_13_H2_C	Healthy	Control	EZTR250005934-1A	9.04	26	0.24	5.1
7	FB_15_H2_C	Healthy	Control	EZTR250005935-1A	9.94	26	0.26	4.6
8	FB_19_H2_C	Healthy	Control	EZTR250005936-1A	5.06	26	0.13	5.2
9	FB_7_I2_RES	Sick	Resveratrol	EZTR250005937-2A	42.13	37	1.56	4.1
10	FB_15_I2_RES	Sick	Resveratrol	EZTR250005939-2A	56.22	37	2.08	4
11	FB_16_I2_RES	Sick	Resveratrol	EZTR250005940-1A	22.35	26	0.58	5.3
12	FB_20_I2_RES	Sick	Resveratrol	EZTR250005941-1A	24.13	26	0.63	5.6
13	FB_22_I2_RES	Sick	Resveratrol	EZTR250005942-1A	19	28	0.53	5.1
14	FB_23_I2_RES	Sick	Resveratrol	EZTR250005943-1A	53.86	28	1.51	5.4
15	FB_4_I2_C	Sick	Control	EZTR250005944-1A	93.38	27	2.52	5.5
16	FB_10_I2_C	Sick	Control	EZTR250005946-1A	36.94	27	1	5.2
17	FB_13_I2_C	Sick	Control	EZTR250005947-1A	64.69	26	1.68	4.7
18	FB_19_I2_C	Sick	Control	EZTR250005948-1A	93.78	26	2.44	7.2
19	FB_27_I2_C	Sick	Control	EZTR250005949-1A	98.4	26	2.56	5.1
20	FB_30_I2_C	Sick	Control	EZTR250005950-1A	69.37	26	1.8	6.3

Table S2.

Available for download at
https://journals.biologists.com/bio/article-lookup/doi/10.1242/bio.062786#supplementary-data

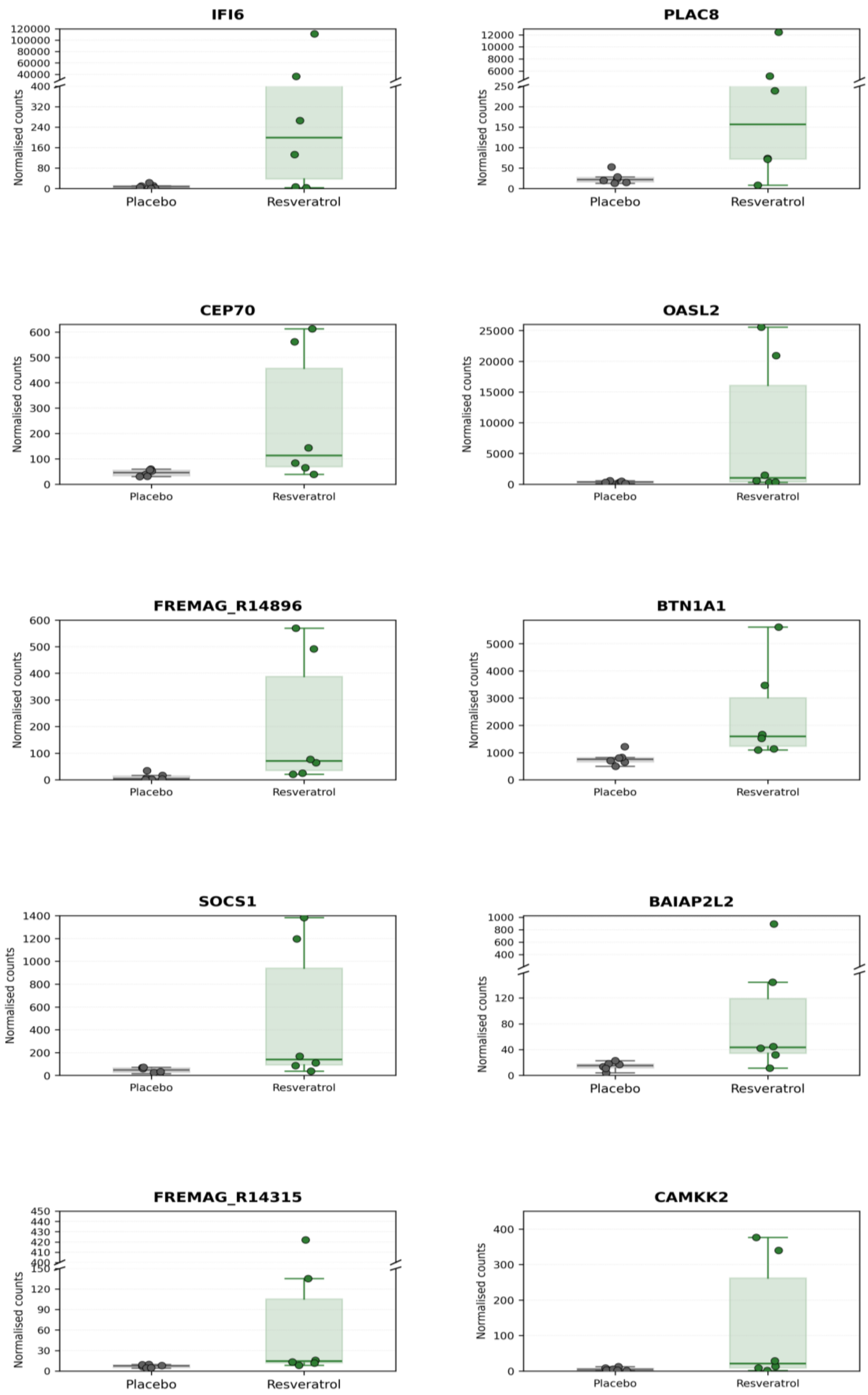

Fig. S1. Dot plots of per-individual normalized counts for DEGs of sick chicks treated with resveratrol (n = 6) and those receiving a placebo (n = 6) in magnificent frigatebird chicks from French Guiana. When needed, Y axes where split in different scales for a better visual representation.

Biology Open • Supplementary information

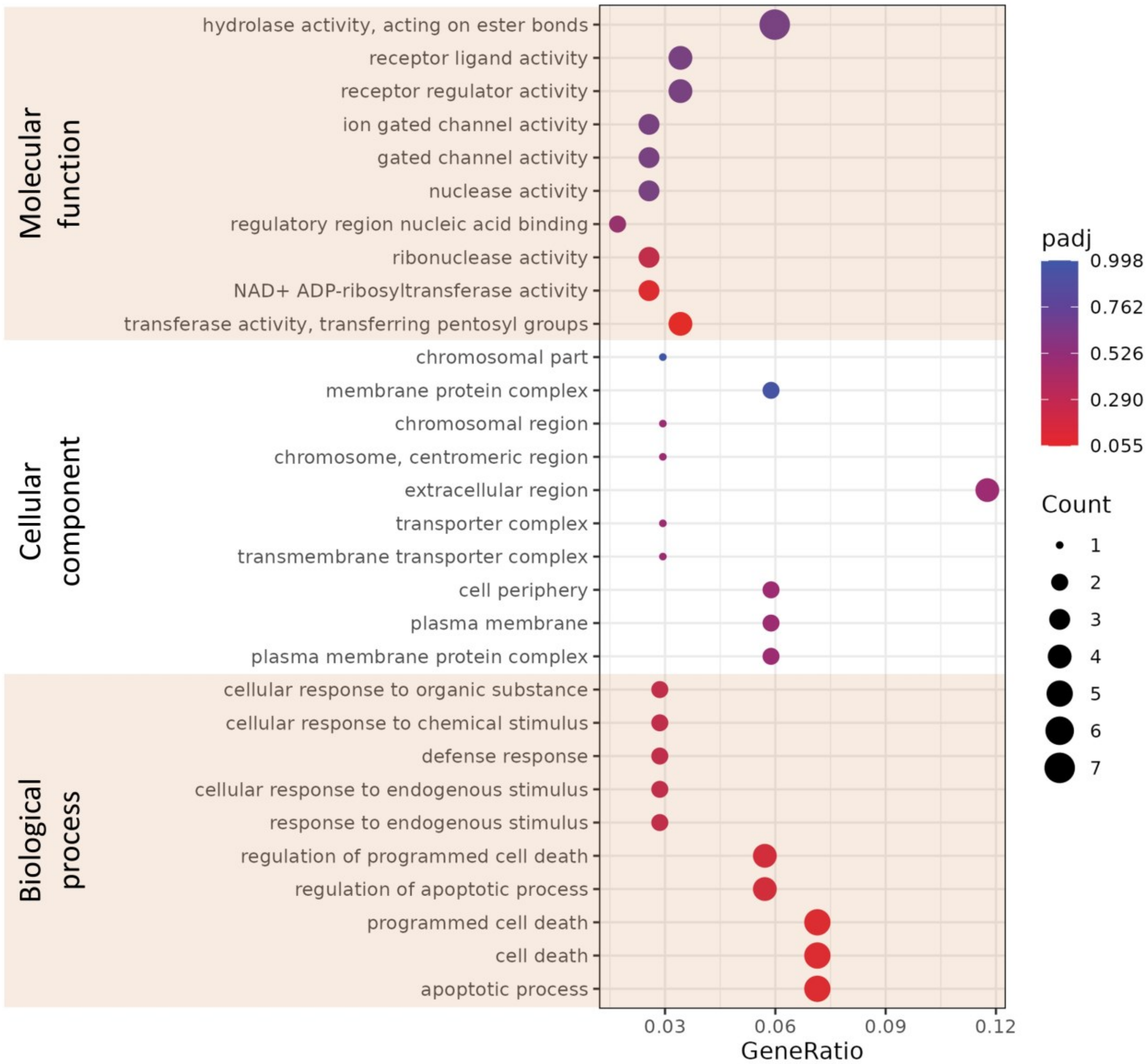

Fig. S2. GO enrichment analyses of DEGs of sick chicks treated with resveratrol (n = 6) and those receiving a placebo (n = 6) in magnificent frigatebird chicks from French Guiana. The first 10 terms of each category (molecular function, cellular component, or biological process) are sorted and coloured by decreasing adjusted p-values, and the size of dots is based on the number of genes corresponding to that term, according to the legend. The Gene ratio refers to the ratio of DEGs over all genes of that specific GO category. Asterisks indicate significant enriched GO terms.

Biology Open • Supplementary information

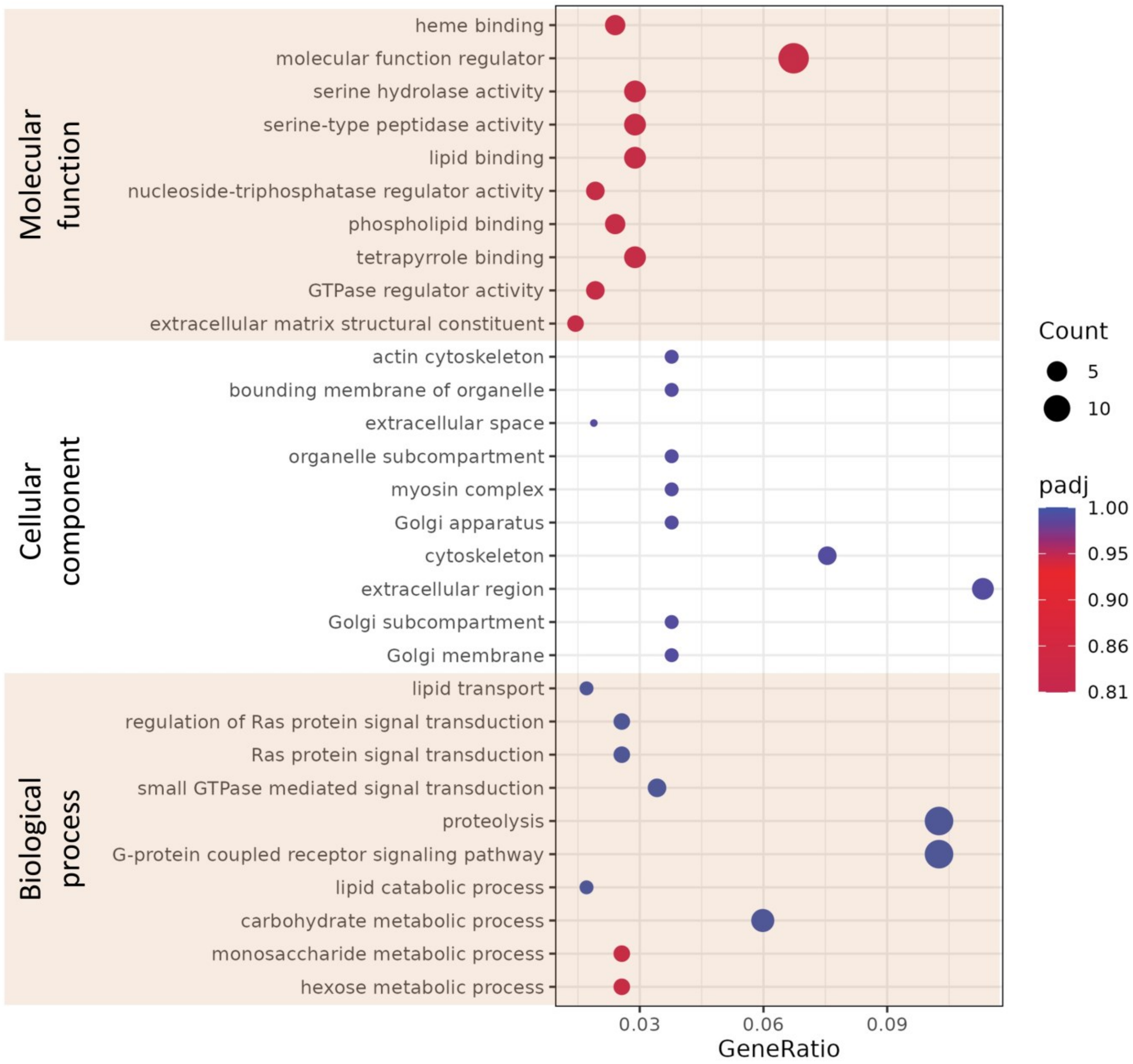

Fig. S3. GO enrichment analyses of DEGs of healthy chicks treated with resveratrol (n = 3) and those receiving a placebo (n = 5) in magnificent frigatebird chicks from French Guiana. The first 10 terms of each category (molecular function, cellular component, or biological process) are sorted and coloured by decreasing adjusted p-values, and the size of dots is based on the number of genes corresponding to that term, according to the legend. The Gene ratio refers to the ratio of DEGs over all genes of that specific GO category. Asterisks indicate significant enriched GO terms.

Biology Open • Supplementary information

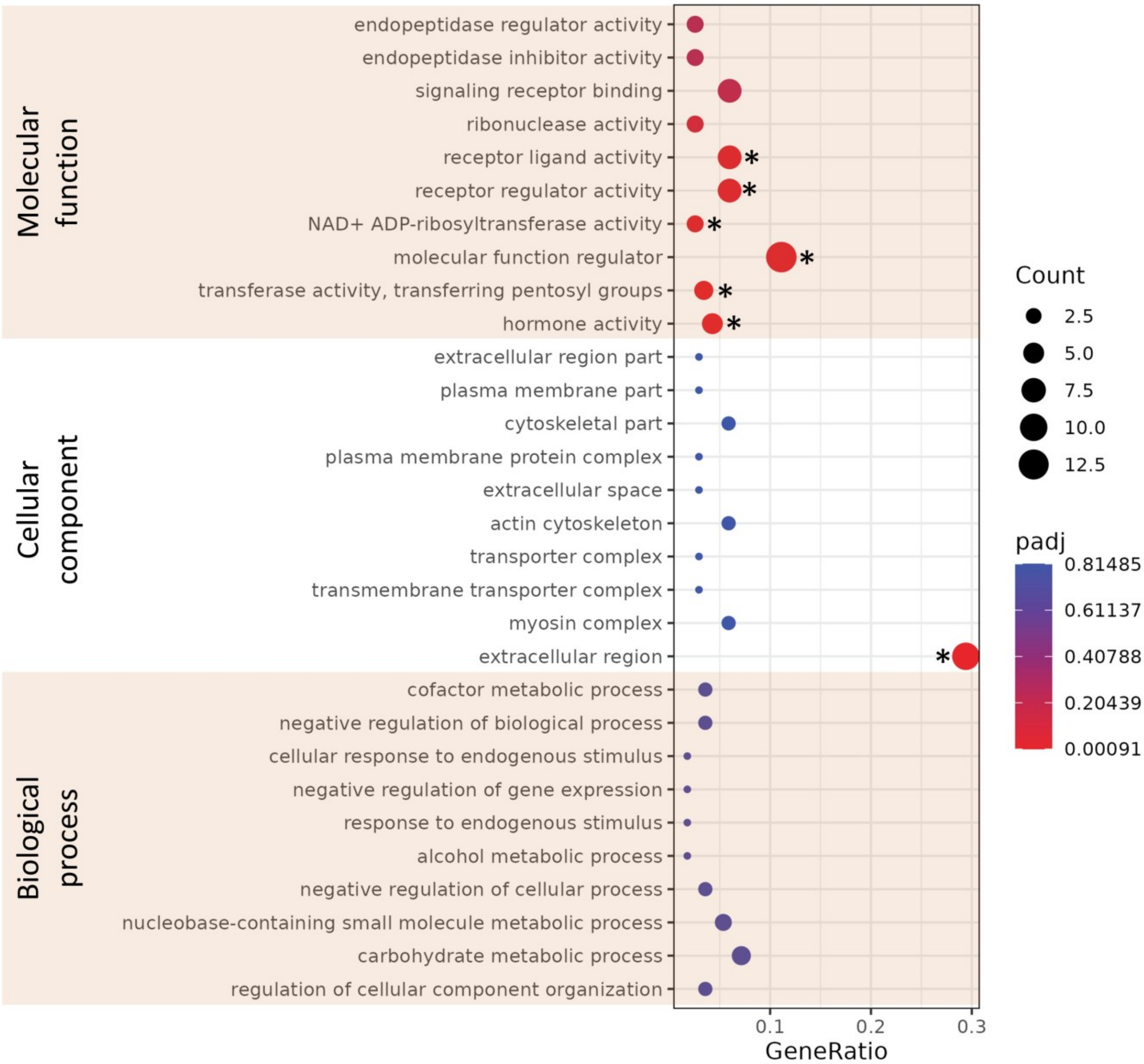

Fig. S4. GO enrichment analyses of DEGs of all chicks treated with resveratrol (n = 9) and those receiving a placebo (n = 11) in magnificent frigatebird chicks from French Guiana, irrespective of their health status. The first 10 terms of each category (molecular function, cellular component, or biological process) are sorted and coloured by decreasing adjusted p-values, and the size of dots is based on the number of genes corresponding to that term, according to the legend. The Gene ratio refers to the ratio of DEGs over all genes of that specific GO category. Asterisks indicate significant enriched GO terms.

Biology Open • Supplementary information

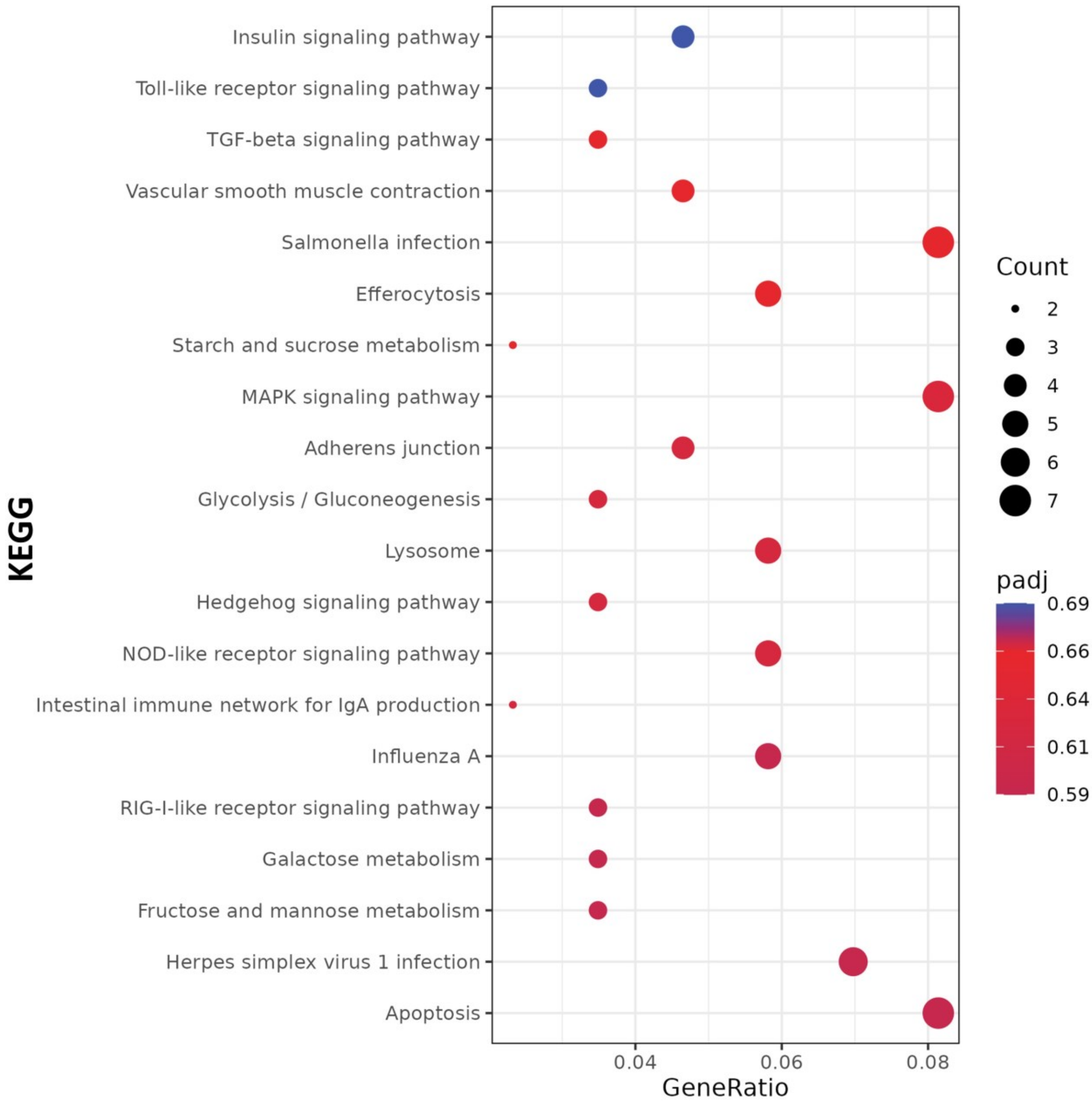

Fig. S5. KEGG enrichment analyses of differential expressed genes in sick magnificent frigatebird chicks from French Guiana that were supplemented with resveratrol (n = 6) or received a placebo (n = 6). KEGG terms are sorted and coloured by decreasing adjusted p-values, and the size of each point is related to the number of genes corresponding to that term, according to the figure legend. The Gene ratio refers to the ratio of differentially expressed genes over all genes of a specific KEGG pathway. Asterisks indicate significantly enriched pathways.

Biology Open • Supplementary information

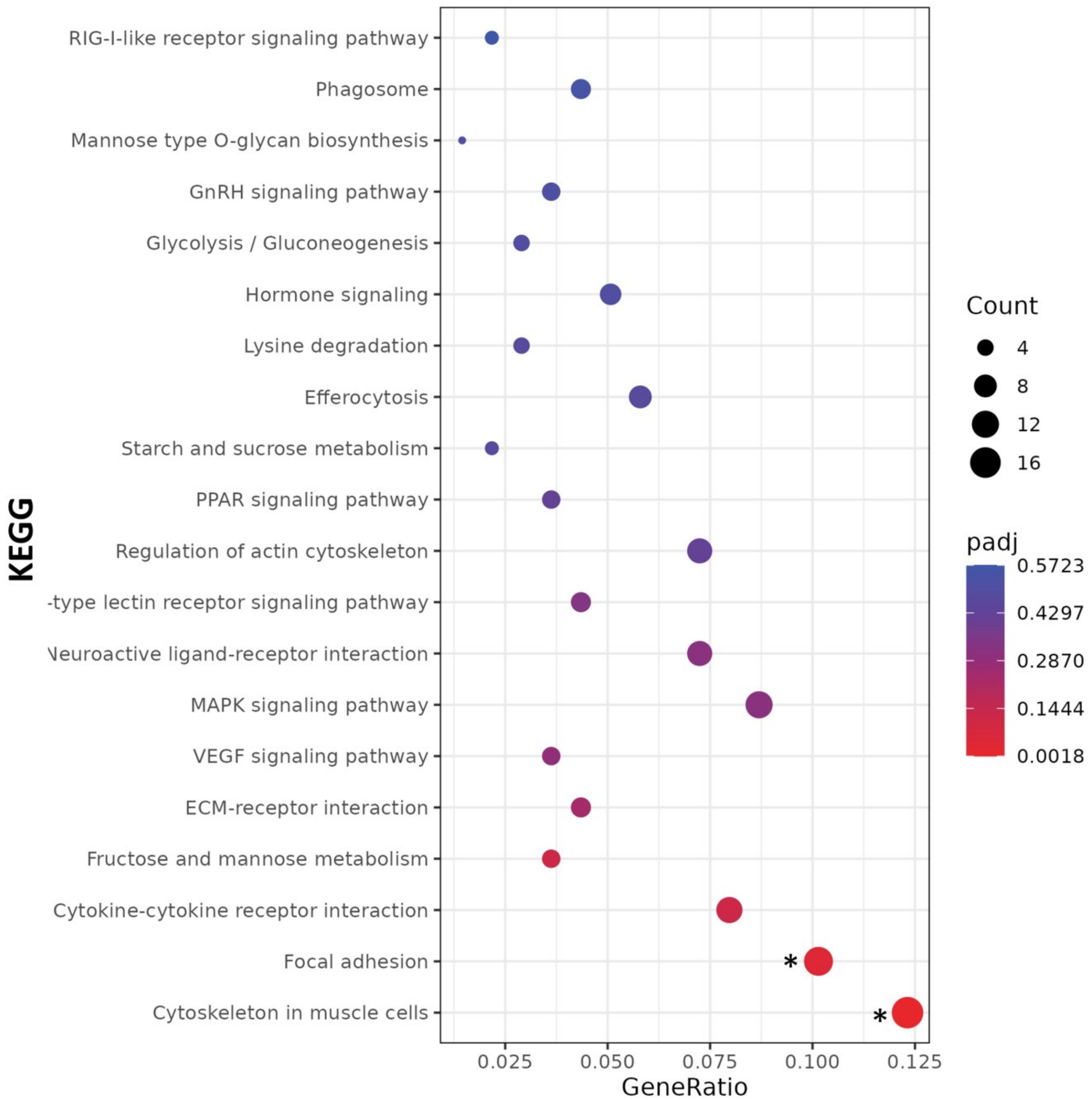

Fig. S6. KEGG enrichment analyses of differential expressed genes in healthy magnificent frigatebird chicks from French Guiana that were supplemented with resveratrol (n = 3) or received a placebo (n = 5). KEGG terms are sorted and coloured by decreasing adjusted p-values, and the size of each point is related to the number of genes corresponding to that term, according to the figure legend. The Gene ratio refers to the ratio of differentially expressed genes over all genes of a specific KEGG pathway. Asterisks indicate significantly enriched pathways.

Biology Open • Supplementary information

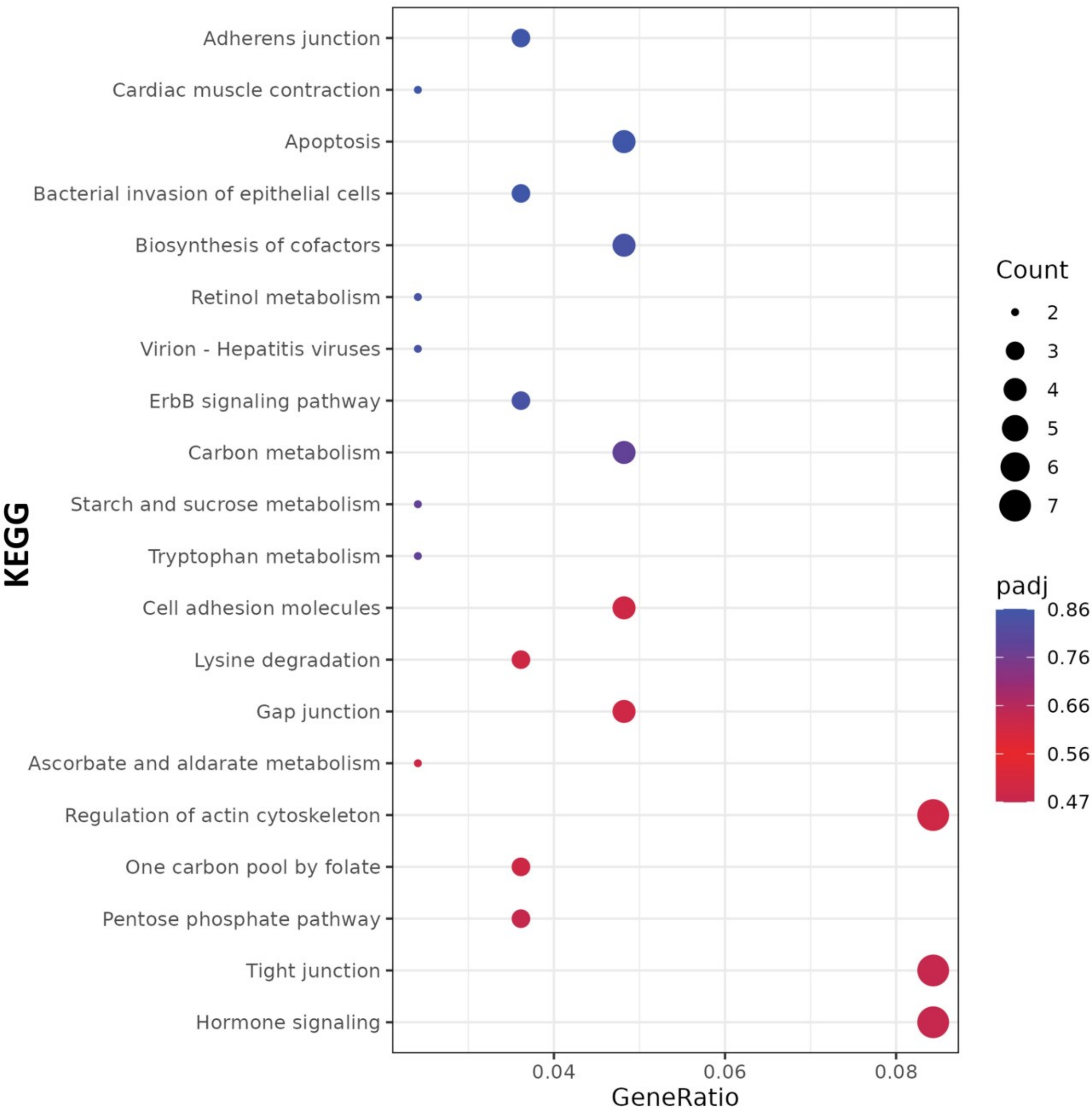

Fig. S7. KEGG enrichment analyses of differential expressed genes in all birds (sick and healthy) magnificent frigatebird chicks from French Guiana that were supplemented with resveratrol (n = 9) or received a placebo (n = 11). KEGG terms are sorted and coloured by decreasing adjusted p-values, and the size of each point is related to the number of genes corresponding to that term, according to the figure legend. The Gene ratio refers to the ratio of differentially expressed genes over all genes of a specific KEGG pathway. Asterisks indicate significantly enriched pathways.

Biology Open • Supplementary information
