# Supplementary material for: Experimental evidence that dietary antioxidants induce an antiviral transcriptomic signature in a wild seabird
Source: Biol Open. 2026 Oct 29;15(10):bio062786. doi: 10.1242/bio.062786 (PMC13750391; doi:10.1242/bio.062786)
Supplement: Supplementary file 2 [file biolopen-15-062786_review_history.pdf]

Experimental evidence that dietary antioxidants induce an antiviral transcriptomic signature in a wild seabird

Manrico Sebastiano, Marcel Eens, Olivier Chastel, Louis Maigné and David Costantini
10.1242/bio.062786

Editor: Valentina di Santo

Review timeline
Original submission: 1 July 2026
Editorial decision: 13 July 2026
First revision received: 11 September 2026
Editorial decision: 18 September 2026
Second revision received: 19 September 2026
Accepted: 21 September 2026

Original submission

First decision letter

MS ID#: bio.062786

MS Title: Experimental evidence that dietary antioxidants induce an antiviral transcriptomic signature in a wild seabird

Authors: Manrico Sebastiano; Marcel Eens; Olivier Chastel; Louis Maigné; David Costantini

I have now reached a decision on the above manuscript.

The reviewer reports are shown at the bottom of this email.

As you will see, the reviewers raised a number of substantial criticisms that prevent me from accepting the paper at this stage.

They suggest, however, that a revised version might prove acceptable, if you can address their concerns. If you think that you can deal satisfactorily with the criticisms on revision, I would be pleased to see a revised manuscript. We would then return it to the reviewers.

At this stage, we also ask you to ensure your manuscript complies with our formatting guidelines. Provided you are able to fully address the referees' comments, we are positive about publication of your paper (we accept over 95% of revision submissions) and therefore hope you won't mind any extra work involved in reformatting your manuscript at this point.

Please upload both a 'clean' version of your Word file, along with a highlighted version clearly showing where you have made changes in the revised manuscript. Please avoid using 'Track changes' in Word files as these are lost in PDF conversion.

I should be grateful if you would also provide a point-by-point response detailing how you have dealt with the points raised by the reviewers in the 'Response to Reviewers' box. Please attend to

© 2026. Published by The Company of Biologists under the terms of the Creative Commons Attribution License (https://creativecommons.org/licenses/by/4.0/).

all of the reviewers' comments. If you do not agree with any of their criticisms or suggestions please explain clearly why this is so.

Reviewer 1

Comments for the author

The manuscript uses whole-blood RNA-seq to test whether dietary resveratrol supplementation reshapes gene expression in magnificent frigatebird (Fregata magnificens) chicks facing a recurrent, herpesvirus-associated disease. I think this is a valuable dataset: a placebo-controlled supplementation experiment carried out in a wild colony, tied to a well-characterised long-term system and to the authors' own earlier work ie Sebastiano et al. 2018. The hypotheses are clearly stated, the placebo control for handling is appropriate, and the ecological framing (resistance vs tolerance) is good. My concerns are not with the concept but with whether the current data and their presentation can carry the strong, causal, mechanistic conclusions drawn from them. Most of my major comments concern statistical power at these sample sizes, the robustness and presentation of the DEGs, reproducibility of the bioinformatics Some claims in the ms go beyond what the transcriptomic data can show. Most my comments can be addressed by re-analysis addign additional supplementary data and wording fixes.

MAJOR COMMENTS

1.Sample sizes and statistical power. The core comparisons rest on very few biological replicates: sick 6 vs 6, healthy 3 vs 5, and pooled 9 vs 11. The healthy contrast (n = 3 resveratrol) is below what is generally considered interpretable for bulk RNA-seq. The benchmarking work of Schurch et al. (2016), using DESeq2 among other tools, recommends at least six replicates per group, rising to ≥12 when DEGs across all fold-change magnitudes matter; with three replicates only ~20-40% of true DEGs are recovered, although recovery exceeds ~85% for genes changing by more than fourfold. The practical consequences for this manuscript are: (i) the strongest, high-fold genes (e.g. IFI6, PLAC8) are the most likely to be real, whereas (ii) the lower-fold DEGs (BTN1A1 ~3-fold, CYGB1 ~3-fold, CEP70 ~5.6-fold) and essentially the entire healthy-chick analysis sit exactly in the regime where both sensitivity and reproducibility are poor. The limitation is currently not acknowledged. I would ask the authors to state the per-group n explicitly at each contrast in the Results (not only in the Table legend), to add a frank power/limitations paragraph to the Discussion, and to be correspondingly cautious about the healthy-chick and KEGG results (see point 6).

2.Extreme fold changes from near-zero counts — show the data spread. Several headline DEGs are driven by counts that are close to the detection limit in one group: IFI6 (sick) 24689 vs 9 normalised reads (2785-fold), PLAC8 2996 vs 26, and in the pooled set IFI6 17001 vs 11 (1574-fold). With 6 (or fewer) replicates, a group mean of ~9-26 reads can be produced by one or two individuals, and DESeq2 log2 fold changes become unstable in this range. Biology Open's guidelines require that graphs let the reader see the true spread of the data. I therefore recommend a supplementary figure showing per-individual normalised counts (dot plots, points per bird) for at least the ten DEGs in Table 1, so readers can judge whether each signal reflects a consistent group difference or a few high-expressing individuals. I would also like to see the use of shrunken effect sizes (e.g. DESeq2 lfcShrink with apeglm; Zhu et al. 2019) for reporting and ranking, since raw LFCs at low counts are exactly what that shrinkage is designed to stabilise. Please also report which genes survived independent filtering and the mean count thresholds applied.

3.Independent validation. Given the small n and the very large fold changes, the transcriptomic signal would be substantially more convincing if a subset of the key DEGs (eg IFI6, OASL2, DDX60, SOCS1) were validated by an orthogonal method such as RT-qPCR on the same or additional individuals. I understand that validation may not be feasible but this should be stated explicitly as a limitation. This is a standard expectation for RNA-seq studies with limited replication (eg Conesa et al. 2016).

4.Reference genome, annotation provenance and gene naming. The bioinformatics are reproducible only in outline. Critically: (i) the reference genome is neither named nor given an accession/version

© 2026. Published by The Company of Biologists under the terms of the Creative Commons Attribution License (https://creativecommons.org/licenses/by/4.0/).

— and it is cited as "(Mortazavi et al., 2008)", which is the RPKM/RNA-seq quantification paper, not a frigatebird genome assembly. Please name the exact assembly (and annotation) used, with its accession and version, and correct this citation. (ii) The gene identifiers (FREMAG_R#####) and the human-readable names carry suffixes (SOCS1_1, OASL2, CEP70_0, BTN1A1_0, CYGB1) that look like automated ortholog/paralog-assignment artefacts. For a non-model seabird this annotation step is central to every biological claim in the paper, yet it is not described: how were FREMAG models assigned gene symbols and functions (reciprocal-best-hit BLAST? against which database and e-value cut-off? OrthoFinder?), and how reliable are the assignments for the genes you interpret? Because much of the Discussion hangs on the identity of ~10 genes, the confidence of each annotation matters and should be reportable (e.g. best-hit species, % identity, e-value) in a supplementary table. (iii) Read QC used "in-house Perl scripts" (Novogene boilerplate) that cannot be reproduced as described — please specify the tool/version or deposit the scripts.

5.Data availability. I could not find a statement on deposition of the raw sequencing reads. As in any transcriptomic study, the raw reads should be deposited in a public archive (NCBI SRA or EMBL-EBI ENA) and the associated BioProject accession cited in the manuscript, together with the gene-level count matrix and the DESeq2/enrichment analysis code (e.g. as a GitHub/Zenodo repository). This is necessary both for reproducibility and to allow readers to inspect the count distributions that underlie the large fold changes (point 2).

6.Enrichment analyses on very small gene sets. GO and KEGG enrichment is being run on DEG lists of 3, 7 and 10 genes. With sets this small, enrichment results are statistically fragile and dominated by one or two genes, and I would ask for the enrichment to be interpreted very conservatively throughout. Two specific issues: (a) the "extracellular region", "hormone activity" and receptor-regulation terms appear only in the pooled analysis and are driven by a mix of up- and down-regulated genes — the biological narrative built around cytokine/hormone signalling should be flagged as exploratory. (b) The healthy-chick KEGG terms "Cytoskeleton in muscle cells" (16 down / 1 up) and "Focal adhesion" (13 down / 1 up) are almost entirely down-regulated, yet they are interpreted as "structural maintenance" and an "investment in host tolerance". A near-uniform down-regulation is difficult to read as increased maintenance investment; either the directionality should be reconciled with the interpretation, or the interpretation softened. (I would also note that "muscle-cell cytoskeleton" and "focal adhesion" terms arising from a whole-blood sample deserve a comment — see next point)

7.Whole-blood cell composition as a confound. Avian whole blood is a heterogeneous, nucleated-cell tissue, and the apparent transcriptome is strongly shaped by the relative proportions of erythrocytes, reticulocytes, heterophils and lymphocytes — which themselves differ with infection and physiological state. Because diseased chicks almost certainly differ from healthy chicks in leukocyte composition (and possibly resveratrol shifts it), some DEGs may reflect shifts in cell-type proportions rather than per-cell regulation. Did the authors have any measure of blood cell composition (e.g. a smear-based differential count, or an in-silico estimate)? At minimum this should be discussed as an alternative explanation, particularly for the structural/cytoskeletal terms.

8.Do the data support "enhance antiviral immunity" and the resistance claim? The central causal claim — that resveratrol enhances antiviral immunity — is inferred entirely from the expression of interferon-stimulated genes; viral load / herpesvirus presence was not measured in the sequenced birds. Elevated ISG expression is equally consistent with resveratrol-treated sick chicks simply carrying a different viral burden, or with among-individual variation in infection stage, especially at these sample sizes. The prediction that responses would be "more pronounced in clinically affected chicks" is also partly confounded with infection state itself (sick chicks have active virus to respond to, irrespective of treatment). I would encourage the authors to (i) temper the title and abstract from a demonstrated increase in "antiviral immunity" toward the transcriptional signature that was actually observed, and (ii) explicitly acknowledge the absence of a viral-load measurement as a limitation for the resistance vs tolerance distinction, which currently reads as more firmly resolved than the data allow.

9.Under-use of the group's own oxidative-stress framework. The mechanistic bridge from "antioxidant intake" to "immune gene expression" is left almost entirely to inference from gene names, with CYGB1 (3-fold) as the only directly cytoprotective/anti-oxidative hit. The authors previously measured circulating antioxidant defences, haptoglobin and lipid oxidative damage in

© 2026. Published by The Company of Biologists under the terms of the Creative Commons Attribution License (https://creativecommons.org/licenses/by/4.0/).

this very experiment (Sebastiano et al. 2018), and state in the Methods that corticosterone, oxidative-stress and immune/inflammatory parameters were measured on the D24 samples. Integrating even the already-published oxidative markers with the transcriptomic groups (e.g. relating individual antioxidant/oxidative-damage values to the ISG response) would materially strengthen the antioxidant→immunity argument and is more in keeping with the mechanistic claim in the title. As it stands, the oxidative-stress mechanism is asserted rather than shown.

STATISTICS

*DESeq2 design formula. The Methods do not give the design formula. Were any covariates included (sex, chick age/beak-head length, RNA quality/RIN, sequencing batch)? Sex in particular is worth checking, as it commonly structures blood transcriptomes; if it could not be modelled given n, please say so.

*"Fold change above 1" wording. The threshold is stated as "adjusted p-values <0.05 and absolute fold change above 1 (or below -1) - corresponding to at least a twofold difference". A fold change of 1 is no change; you mean |log2 fold change| > 1. Please correct the wording to log2FC to match Table 1's columns.

*Multiple contrasts. Three separate DESeq2 contrasts (sick, healthy, pooled) are run and interpreted. The pooled result largely recapitulates the sick result (as the authors note, sick chicks are >50% of the pool); it would help to state clearly that the pooled contrast is not independent evidence.

*Report the number of genes tested and the independent-filtering outcome for each contrast, so the reader can judge the multiple-testing burden behind the padj values.

MINOR COMMENTS

*Table 1: the two "NA"/novel transcripts (FREMAG_R14896, FREMAG_R14315) are counted within the "10 DEGs" in sick chicks but excluded from interpretation. Please state the DEG counts consistently (e.g. "10 DEGs, of which 2 are unannotated novel transcripts") wherever the number is used.

*Table 1 / text: DDX60 and CYGB1 are described as "upregulated by 7 and 3 folds"; please make sure the fold values quoted in the text match the Fold-change column of Table 1 throughout, and give the direction consistently.

*Gene-name spelling: "OASL2 (2'-5'-Oligoadenilato Sintetasi 2)" uses the Italian spelling; the standard English is "2'-5'-oligoadenylate synthetase-like 2". Please standardise gene descriptions to the official symbols/names (e.g. HGNC), and clarify the "_0"/"_1" suffixes (point 4-ii).

*Citation for the reference genome: replace "(Mortazavi et al., 2008)" for genome alignment with the correct genome assembly reference (point 4-i). Also double-check that the intended aligner citation (HISAT2, Kim et al.) is the one attached to "Hisat2 v2.0.5".

*Methods, sample selection: the RNA-seq subset is "20 birds with clear health history" drawn from 60. Please state the selection criteria and confirm these 20 are representative (e.g. not selected on disease severity or RNA yield), as non-random selection would bias the treatment contrasts.

*Please report RNA integrity (RIN) values per sample (e.g. in the supplement), and confirm whether any samples were excluded on quality grounds. Read depth per sample and mapping rates would also be useful for assessing rigour.

*Methods say plasma was used for corticosterone, oxidative-stress and immune/inflammatory parameters; since none are reported here, either integrate them (point 9) or cross-reference where they are/will be reported, so the reader is not left expecting them.

*"CYGB1" — cytoglobin is normally CYGB; please confirm the "1" is a genuine paralog and not an annotation suffix, since it is used as a substantive cytoprotection argument.

© 2026. Published by The Company of Biologists under the terms of the Creative Commons Attribution License (https://creativecommons.org/licenses/by/4.0/).

*Consider stating the herpesvirus context more carefully: the disease is described as herpesvirus-associated (de Thoisy et al. 2009) but not confirmed in the study birds; wording such as "likely actively replicating" should be flagged as an assumption.

REFERENCES

Conesa A, Madrigal P, Tarazona S, Gomez-Cabrero D, Cervera A, McPherson A, et al. (2016). A survey of best practices for RNA-seq data analysis. Genome Biology 17: 13. doi:10.1186/s13059-016-0881-8

Costantini D (2022). A meta-analysis of impacts of immune response and infection on oxidative status in vertebrates. Conservation Physiology 10: coac018. (cited in the manuscript)

Schurch NJ, Schofield P, Gierliński M, Cole C, Sherstnev A, Singh V, et al. (2016). How many biological replicates are needed in an RNA-seq experiment and which differential expression tool should you use? RNA 22: 839-851. doi:10.1261/rna.053959.115

Sebastiano M, et al. (2018). [Resveratrol supplementation, antioxidant defences, haptoglobin and lipid oxidative damage in frigatebird chicks — as cited in the manuscript.]

Zhu A, Ibrahim JG, Love MI (2019). Heavy-tailed prior distributions for sequence count data: removing the noise and preserving large differences. Bioinformatics 35: 2084-2092. doi:10.1093/bioinformatics/bty895

I used an AI assistant only to help organise, structure and copy-edit my own review notes and to look up methodological references; the assessment and conclusions are my own.

<u>Reviewer 2</u>

Comments for the author

This is an interesting study examining the impact of resveratrol supplementation on gene expression changes in healthy and sick Magnificent frigatebirds, and its potential impact on immune health in this species.

This review was carried out as per the BIO-OPEN rubric, alongside some specific further comments made at the end:

1.Experimental quality

a.Does each figure have the proper controls?

All figures appear to use the correct controls for the type of data shown.

b.Are experiments performed using appropriate methods that will answer the question (or test the hypothesis or support the observations) posed by the authors? Is the right tool used for the job?

Yes

c.Were the data analysed using appropriate statistical tests?

Yes

2.Reproducibility

a.Were experiments in each figure performed using adequate number of biological replicates?

© 2026. Published by The Company of Biologists under the terms of the Creative Commons Attribution License (https://creativecommons.org/licenses/by/4.0/).

Yes.

b.Is there sufficient raw data to assess the rigor of the analysis?

No.
It is standard practice to deposit raw transcriptome sequencing data in a public sequencing repository, for example, the NCBI Sequence Read Archive under a BioProject accession number. Then interested readers can analyse the data themselves.

There has been an attempt to include the results of the DEG analysis on PDF pages 19-66. I think this includes the background gene set used for KEGG enrichment; however, it is extremely difficult to interpret. It appears as though Excel data has been pasted into the Word format, so you have connected information spanning several pages of the manuscript. It would be far better to provide this information as a supplementary table in Excel.

c.Does the methods section provide sufficient detail to permit reproducibility?

Not fully. Methods requiring more details are:

No details are given on the blood processing and RNA isolation method (line 155).

The methods jump from library preparation (lines 158-160) straight to sequence read filtering/mapping/analysis. There is no mention of how the sequencing was done. Please add this information.

Line 160: "real-time PCR for quantification and bioanalyzer". Neither of these methods have sufficient detail provided to permit reproducibility. For "bioanalyzer", do you mean an Agilent bioanalyser? So, a different analyser to the 5400 fragment analyser indicated on line 155?

Lines 182-191: In the Methods subsection "Enrichment analyses of differentially expressed genes", please specify all the analysis tools, databases, and software packages used.

3.Completeness

a.Are the author's conclusions supported by the data?

Generally, yes. However, the discussion centred around the specific DEGs identified (lines 297-348) should make clear that this is a relatively small group of genes. Therefore, interpretations should be cautious, and conclusions drawn can only be validated by repeating the experiment alongside further, complementary, experiments. Or words to this effect.

The manuscript title has "enhance antiviral immunity", which feels quite a bold description. I would prefer something a bit more toned down to indicate resveratrol affects the expression of genes with known roles in antiviral immunity.

b.Are there any flaws in the experimental design that invalidate the approach taken by the authors?

Nothing obvious to me. The experimental design appears to be robust.

I am interested to know how simple/challenging capturing the birds for repeated sampling is?

c.Are there experiments that have not been performed, but if true would disprove the conclusion? If yes, and if such experiments would be costly or time-consuming to perform, do the authors acknowledge this in a discussion of the limitations?

© 2026. Published by The Company of Biologists under the terms of the Creative Commons Attribution License (https://creativecommons.org/licenses/by/4.0/).

Additional experiments that could be performed and are not mentioned in the discussion (they would be costly):

Single-cell transcriptomics on PBMCs to determine which subpopulations exhibit the greatest transcriptional response. This may identify other DEGs masked by the bulk sequencing approach.

Combining transcriptome analysis with proteomics (a good method of validating the biological relevance of the highly expressed genes identified).

A few sentences could be added to acknowledge these.

4.Scholarship

a.Do the authors cite and discuss the merits of relevant data that would argue against their conclusion?

No, however, the points they discuss are relevant and clear and appropriately cited. The authors also included this in the discussion "However, because these patterns emerged only in pooled analyses, future studies with larger sample sizes and longitudinal designs will be necessary to determine whether these transcriptional shifts represent early regulatory responses that precede phenotypic changes in disease progression". Which I feel adequately acknowledges the limitation of the findings.

b.Do the authors cite and discuss the merits of relevant data that would support their conclusion?

Yes, discussion points are relevant and clear and appropriately cited.

c.For techniques/methods manuscripts, Do the authors cite and discuss the current state of the field and clearly explain how the method improves the field?

Not relevant with this manuscript.

Specific points that require attention:

Line 157-160 - was a specific kit used for the library prep?

Lines 165-166: "All the downstream analyses were based on the clean data with high quality". The English could be improved here. For example. "All downstream analyses used only high quality clean data".

Line 202-203: You excluded two (highly expressed) unannotated transcripts. You later (on line 219), mention this was because one of these (FREMAG_R14896) lacked known orthologues. Was this also the case with FREMAG_R14315?

Line 343: please remove the word "significant".

When describing fold changes in expression, the plural form of the word "folds" is used repeatedly. The singular form "fold" is the correct one to use. Please amend.

Was ethical approval required for this study? If so, details are missing. Please specify or clearly indicate which reference has this information.

Did the authors consider carrying out something like weighted gene co-expression network analysis (WGCNA)? Or a different type of gene regulatory network analysis? Or would it not have added much and been largely speculative with this data set?

Reviewer's Responses to Questions

© 2026. Published by The Company of Biologists under the terms of the Creative Commons Attribution License (https://creativecommons.org/licenses/by/4.0/).

Experimental quality
Are experiments performed using appropriate methods that will answer the question (or test the hypothesis or support the observations) posed by the authors? Is the right tool used for the job?
If 'No', please indicate reasons in Comments for Author box below.
Reviewer #1:
- Yes

Reviewer #2:
- Yes

Were the data analyzed using appropriate statistical tests?
If 'No', please indicate reasons in Comments for Author box below.
Reviewer #1:
- Yes

Reviewer #2:
- Yes

Reproducibility
Were experiments performed using adequate number of biological replicates?
If 'No', please indicate reasons in Comments for Author box below.
Reviewer #1:
- No

Reviewer #2:
- Yes

Is there sufficient raw data to assess the rigor of the analysis?
If 'No', please indicate reasons in Comments for Author box below.
Reviewer #1:
- No

Reviewer #2:
- No

Does the methods section provide sufficient detail to permit reproducibility?
If 'No', please indicate reasons in Comments for Author box below.
Reviewer #1:
- No

Reviewer #2:
- No

Completeness
Are the manuscript's conclusions supported by the data?
If 'No', please indicate reasons in Comments for Author box below.
Reviewer #1:
- No

Reviewer #2:
- Yes

Scholarship
Do the authors cite and discuss the merits of data that would argue for and against their conclusion?
If 'No', please indicate reasons in Comments for Author box below.
Reviewer #1:
- Yes

Reviewer #2:
- No

Does the manuscript title & abstract accurately reflect the contents of the manuscript, without hyperbole?
If 'No', please indicate reasons in Comments for Author box below.

© 2026. Published by The Company of Biologists under the terms of the Creative Commons Attribution License (https://creativecommons.org/licenses/by/4.0/).

Reviewer #1:
- No

Reviewer #2:
- No

First revision

Author response to reviewers' comments

Submission of manuscript ID bio.062786 after revision

Dear Editor,

Please, find enclosed a revised version of our manuscript now entitled "Experimental evidence that dietary antioxidants induce an antiviral transcriptomic signature in a wild seabird" by myself, Marcel Eens, Olivier Chastel, Louis Maigné, and David Costantini, submitted for reconsideration for publication in Biology Open.

We would like to thank you and the reviewers for the time and effort devoted to evaluating our manuscript and for the constructive comments and suggestions provided. We have carefully considered all remarks and have revised the manuscript accordingly. Below, we provide a detailed, point-by-point response to each comment. Our responses are indicated in bold and preceded by the abbreviation "A".

We believe that the revisions have substantially strengthened the manuscript and clarified several important aspects of the study. We hope that the revised version will now be suitable for publication in Biology Open and will be of interest to the journal's readership.

Reviewer 1

Reviewer 1: The manuscript uses whole-blood RNA-seq to test whether dietary resveratrol supplementation reshapes gene expression in magnificent frigatebird (Fregata magnificens) chicks facing a recurrent, herpesvirus-associated disease. I think this is a valuable dataset: a placebo-controlled supplementation experiment carried out in a wild colony, tied to a well-characterised long-term system and to the authors' own earlier work ie Sebastiano et al. 2018. The hypotheses are clearly stated, the placebo control for handling is appropriate, and the ecological framing (resistance vs tolerance) is good. My concerns are not with the concept but with whether the current data and their presentation can carry the strong, causal, mechanistic conclusions drawn from them. Most of my major comments concern statistical power at these sample sizes, the robustness and presentation of the DEGs, reproducibility of the bioinformatics Some claims in the ms go beyond what the transcriptomic data can show. Most my comments can be addressed by re-analysis addign additional supplementary data and wording fixes.

MAJOR COMMENTS

1.Sample sizes and statistical power. The core comparisons rest on very few biological replicates: sick 6 vs 6, healthy 3 vs 5, and pooled 9 vs 11. The healthy contrast (n = 3 resveratrol) is below what is generally considered interpretable for bulk RNA-seq. The benchmarking work of Schurch et al. (2016), using DESeq2 among other tools, recommends at least six replicates per group, rising to ≥12 when DEGs across all fold-change magnitudes matter; with three replicates only ~20-40% of true DEGs are recovered, although recovery exceeds ~85% for genes changing by more than fourfold. The practical consequences for this manuscript are: (i) the strongest, high-fold genes (e.g. IFI6, PLAC8) are the most likely to be real, whereas (ii) the lower-fold DEGs (BTN1A1 ~3-fold, CYGB1 ~3-fold, CEP70 ~5.6-fold) and essentially the entire healthy-chick analysis sit exactly in the regime where both sensitivity and reproducibility are poor. The limitation is currently not acknowledged. I would ask the authors to state the per-group n explicitly at each contrast in the

© 2026. Published by The Company of Biologists under the terms of the Creative Commons Attribution License (https://creativecommons.org/licenses/by/4.0/).

Results (not only in the Table legend), to add a frank power/limitations paragraph to the Discussion, and to be correspondingly cautious about the healthy-chick and KEGG results (see point 6).
A. We thank the reviewer for this important point. We agree that the healthy-chick contrast (n = 3 vs 5) is underpowered by conventional RNA-seq benchmarks, and we have now stated the per-group sample sizes explicitly in the Results text for each of the three contrasts (not only in the Table 1 legend). We have also added a dedicated paragraph to the Discussion acknowledging the power limitations of the healthy-chick and pooled analyses, following Schurch et al. (2016), and we now discuss the lower-fold DEGs (BTN1A1, CYGB1, CEP70) and the healthy-chick/KEGG results as provisional pending confirmation in a larger sample.

The following paragraph was included in the main text:
"Finally, we would like to point out that a key limitation of this study is the small number of biological replicates underlying the DESeq2 contrasts, particularly for the healthy-chick comparison (n = 3 resveratrol vs 5 placebo). Benchmarking work by Schurch et al. (2016) showed that at three replicates per group, RNA-seq differential expression analyses recover only ~20-40% of true DEGs overall. Under this framework, the lower-fold DEGs identified in this study as BTN1A1 (~3-fold), CYGB1 (~3-fold), and CEP70 (~5.6-fold), fall within a range where both sensitivity and reproducibility are expected to be low at these sample sizes. We therefore interpret these three genes with caution, and note that they should be treated as provisional until confirmed in a larger sample. The healthy-chick contrast as a whole, including its associated KEGG enrichment results (cytoskeleton in muscle cells, focal adhesion), sits below the replicate number generally considered sound and should accordingly be regarded as exploratory rather than conclusive. Future studies should i) consider larger sample sizes, particularly for the healthy-chick comparison, to confirm these lower-magnitude transcriptional responses, and ii) validate of DEGs via real-time qPCR to increase confidence in the interpretation of biological responses (Conesa et al. 2016)."

2.Extreme fold changes from near-zero counts — show the data spread. Several headline DEGs are driven by counts that are close to the detection limit in one group: IFI6 (sick) 24689 vs 9 normalised reads (2785-fold), PLAC8 2996 vs 26, and in the pooled set IFI6 17001 vs 11 (1574-fold). With 6 (or fewer) replicates, a group mean of ~9-26 reads can be produced by one or two individuals, and DESeq2 log2 fold changes become unstable in this range. Biology Open's guidelines require that graphs let the reader see the true spread of the data. I therefore recommend a supplementary figure showing per-individual normalised counts (dot plots, points per bird) for at least the ten DEGs in Table 1, so readers can judge whether each signal reflects a consistent group difference or a few high-expressing individuals. I would also like to see the use of shrunken effect sizes (e.g. DESeq2 lfcShrink with apeglm; Zhu et al. 2019) for reporting and ranking, since raw LFCs at low counts are exactly what that shrinkage is designed to stabilise. Please also report which genes survived independent filtering and the mean count thresholds applied.
A. We thank the reviewer for the suggestion. We have now provide a dot plot graph in the supplementary information and we refer to it within the manuscript. We re-ran the differential expression analyses for all three contrasts (sick chicks, healthy chicks, and the pooled dataset) using DESeq2's lfcShrink function with the apeglm estimator a suggested (Zhu et al., 2019), and Tables 1 and 2 now report shrunken log2 fold changes throughout in place of raw maximum-likelihood estimates.
In the sick-chick contrast, shrinkage reduced the fold-change estimates for all ten DEGs by approximately 13-37% (e.g., Ifi6: 2784-fold → 1924-fold; Plac8: 117-fold → 85-fold), while padj values and gene ranking were unaffected, indicating that these effects are not artifacts of unstable low-count estimation but are moderated, more conservative effect sizes.
In the healthy-chick contrast, applying the same procedure confirmed our decision to exclude Col4a1 from interpretation: this gene's raw log2 fold change (driven by the absence of detectable expression in the majority of samples) collapsed to approximately zero after shrinkage. The two retained healthy-chick DEGs, Sptbn4 and Entpd3, showed slightly shrunken fold changes of ~4.7-fold (down) and ~5.3-fold (up), respectively (previously ~6-fold and ~7-fold).
In the pooled contrast, five of the seven DEGs showed the expected reduction in fold-change magnitude after shrinkage (e.g., Cygb1: 2.9-fold → 2.5-fold; Cep70: 4.5-fold → 4.0-fold). Two genes, Ifi6 and Plac8, even showed a modest increase in shrunken fold change relative to the raw estimate (398.9-fold → 1266.6-fold and 23.7-fold → 64.3-fold, respectively).

© 2026. Published by The Company of Biologists under the terms of the Creative Commons Attribution License (https://creativecommons.org/licenses/by/4.0/).

We have also added the independent filtering results (mean count thresholds and numbers of genes tested/excluded per contrast) to the Methods, as detailed below."

The following sentences were included in the methods:
"Log2 fold changes were shrunk using the apeglm estimator (Zhu et al., 2019) as implemented in DESeq2's lfcShrink function, and shrunken values are reported throughout in place of raw maximum-likelihood estimates. Note that the pooled contrast (resveratrol-supplemented vs. control birds, irrespective of health status) is not statistically independent of the sick-chick contrast, since sick chicks make up >50% of the pooled sample.
Independent filtering and outlier exclusion (Cook's distance) were applied separately per contrast. In the sick-chick contrast, of 11,633 genes with nonzero counts, 88 (0.76%) were excluded as count outliers and 2,913 (25%) by independent filtering (mean count threshold = 4), leaving 8,632 genes tested. In the healthy-chick contrast, of 11,611 genes, 5 (0.043%) were excluded as outliers and none by independent filtering (threshold = 0); one further gene, Col4a1, was excluded from interpretation despite meeting nominal significance and fold-change thresholds, as it showed no detectable expression in most samples (zero counts in all three resveratrol-treated birds and three of five controls) — apeglm shrinkage collapsed its log2 fold change to approximately zero, consistent with this being a numerical artifact rather than a genuine effect. In the pooled contrast, of 12,264 genes, none were excluded as outliers and 4,999 (41%) were excluded by independent filtering (threshold = 15), leaving 7,265 genes tested. All tests were corrected for multiple comparisons using the Benjamini-Hochberg method."

3.Independent validation. Given the small n and the very large fold changes, the transcriptomic signal would be substantially more convincing if a subset of the key DEGs (eg IFI6, OASL2, DDX60, SOCS1) were validated by an orthogonal method such as RT-qPCR on the same or additional individuals. I understand that validation may not be feasible but this should be stated explicitly as a limitation. This is a standard expectation for RNA-seq studies with limited replication (eg Conesa et al. 2016).
A. We agree that orthogonal validation (e.g. RT-qPCR) would strengthen confidence in the key DEGs, but we were unable to carry it out. We have included this limitation in the main text.

The following sentences was included in the discussion:
"Future studies should i) consider larger sample sizes, particularly for the healthy-chick comparison, to confirm these lower-magnitude transcriptional responses, and ii) validate of DEGs via real-time qPCR to increase confidence in the interpretation of biological responses (Conesa et al., 2016)."

4.Reference genome, annotation provenance and gene naming. The bioinformatics are reproducible only in outline. Critically: (i) the reference genome is neither named nor given an accession/version — and it is cited as "(Mortazavi et al., 2008)", which is the RPKM/RNA-seq quantification paper, not a frigatebird genome assembly. Please name the exact assembly (and annotation) used, with its accession and version, and correct this citation. (ii) The gene identifiers (FREMAG_R#####) and the human-readable names carry suffixes (SOCS1_1, OASL2, CEP70_0, BTN1A1_0, CYGB1) that look like automated ortholog/paralog-assignment artefacts. For a non-model seabird this annotation step is central to every biological claim in the paper, yet it is not described: how were FREMAG models assigned gene symbols and functions (reciprocal-best-hit BLAST? against which database and e-value cut-off? OrthoFinder?), and how reliable are the assignments for the genes you interpret? Because much of the Discussion hangs on the identity of ~10 genes, the confidence of each annotation matters and should be reportable (e.g. best-hit species, % identity, e-value) in a supplementary table. (iii) Read QC used "in-house Perl scripts" (Novogene boilerplate) that cannot be reproduced as described — please specify the tool/version or deposit the scripts.
A: We thank the reviewer for these important points regarding reproducibility.
(i) The reviewer is correct that the citation attached to the reference genome was incorrect; Mortazavi et al. (2008) describe the RPKM normalisation method, not a genome assembly. Reads were aligned to the *Fregata magnificens* genome assembly ASM1338994v1 (GenBank accession GCA_013389945.1), assembled with SOAPdenovo v2.04 (~40x Illumina HiSeq coverage) as part of the Bird 10,000 Genomes (B10K) Project, Family phase (Feng et al., 2020, *Nature*). We have corrected the citation and added the assembly accession and method to the Methods.

© 2026. Published by The Company of Biologists under the terms of the Creative Commons Attribution License (https://creativecommons.org/licenses/by/4.0/).

(ii) Gene identifiers and symbols were assigned by the B10K project's comparative annotation pipeline (Feng et al., 2020), which projects gene models onto each genome via a reference-free whole-genome alignment (Cactus) using conserved synteny with the chicken (*Gallus gallus*) reference annotation, rather than a similarity-search approach. Numeric suffixes (e.g., _0, _1) are appended by this pipeline when multiple loci are projected onto the same base gene symbol. We have added this description to the Methods. We are unable to provide gene-by-gene synteny support scores for the discussed genes, as these are not reported by the annotation provider beyond what is included in the publicly available annotation files.
(iii) We agree that "in-house Perl scripts" were insufficiently specific. Raw reads were quality-filtered by removing adapter sequences, poly-N reads, and low-quality reads to obtain clean data, and Q20/Q30 and GC content were calculated for quality assessment. We have updated the Methods accordingly.

REFERENCE: Feng, S., Stiller, J., Deng, Y. *et al*. Dense sampling of bird diversity increases power of comparative genomics. *Nature* 587, 252–257 (2020). https://doi.org/10.1038/s41586-020-2873-9

The following sentence was included in the methods:
"Reads were aligned to the Fregata magnificens reference genome assembly ASM1338994v1 (GenBank accession GCA_013389945.1; SOAPdenovo v2.04, ~40x Illumina HiSeq coverage), generated as part of the Bird 10,000 Genomes (B10K) Project (Feng et al., 2020), using Hisat2 v2.0.5 (Kim et al., 2019). Gene identifiers and symbols follow the B10K project's comparative annotation, in which gene models are projected onto each genome via a reference-free whole-genome alignment (Cactus) using conserved synteny with the chicken (Gallus gallus) reference annotation (Feng et al., 2020); numeric suffixes (e.g., _0, _1) indicate multiple loci projected onto the same base gene symbol."

5.Data availability. I could not find a statement on deposition of the raw sequencing reads. As in any transcriptomic study, the raw reads should be deposited in a public archive (NCBI SRA or EMBL-EBI ENA) and the associated BioProject accession cited in the manuscript, together with the gene-level count matrix and the DESeq2/enrichment analysis code (e.g. as a GitHub/Zenodo repository). This is necessary both for reproducibility and to allow readers to inspect the count distributions that underlie the large fold changes (point 2).
A. We thank the reviewer for raising this important point. The raw sequencing reads for all 20 samples have been deposited in the NCBI Sequence Read Archive under BioProject accession PRJNA1519313. The gene-level count matrices are provided as a supplementary Excel table (Table Sx), and the DESeq2 analysis workflow (including the design formula, filtering, and shrinkage procedure) is fully described in the Methods, allowing readers to inspect the count distributions underlying the reported fold changes. We have added a Data Availability statement to the manuscript.

6.Enrichment analyses on very small gene sets. GO and KEGG enrichment is being run on DEG lists of 3, 7 and 10 genes. With sets this small, enrichment results are statistically fragile and dominated by one or two genes, and I would ask for the enrichment to be interpreted very conservatively throughout. Two specific issues: (a) the "extracellular region", "hormone activity" and receptor-regulation terms appear only in the pooled analysis and are driven by a mix of up- and down-regulated genes — the biological narrative built around cytokine/hormone signalling should be flagged as exploratory. (b) The healthy-chick KEGG terms "Cytoskeleton in muscle cells" (16 down / 1 up) and "Focal adhesion" (13 down / 1 up) are almost entirely down-regulated, yet they are interpreted as "structural maintenance" and an "investment in host tolerance". A near-uniform down-regulation is difficult to read as increased maintenance investment; either the directionality should be reconciled with the interpretation, or the interpretation softened. (I would also note that "muscle-cell cytoskeleton" and "focal adhesion" terms arising from a whole-blood sample deserve a comment — see next point).
A. We agree that enrichment results from DEG lists of 3-10 genes are not very robust statistically speaking, and we have added a paragraph stating that GO/KEGG results should be interpreted as exploratory given the small input gene sets, as requested. Regarding the healthy-chick KEGG terms ('Cytoskeleton in muscle cells', 'Focal adhesion'), we agree that most genes were downregulated thus difficult to reconcile with our original framing of 'structural maintenance' as increased investment in tolerance. We have revised this interpretation in the

© 2026. Published by The Company of Biologists under the terms of the Creative Commons Attribution License (https://creativecommons.org/licenses/by/4.0/).

Discussion to note that predominant downregulation more plausibly reflects reduced structural/cytoskeletal investment, rather than framing it as a positive tolerance mechanism.

The following sentence was included:
As a predominant downregulation of genes was observed (16/17 and 13/14 genes downregulated), this more plausibly reflects reduced structural/cytoskeletal investment, rather than a strengthening of tissue barriers against future pathogen-induced damage (Ayres and Schneider, 2012) as hypothesized.

7.Whole-blood cell composition as a confound. Avian whole blood is a heterogeneous, nucleated-cell tissue, and the apparent transcriptome is strongly shaped by the relative proportions of erythrocytes, reticulocytes, heterophils and lymphocytes — which themselves differ with infection and physiological state. Because diseased chicks almost certainly differ from healthy chicks in leukocyte composition (and possibly resveratrol shifts it), some DEGs may reflect shifts in cell-type proportions rather than per-cell regulation. Did the authors have any measure of blood cell composition (e.g. a smear-based differential count, or an in-silico estimate)? At minimum this should be discussed as an alternative explanation, particularly for the structural/cytoskeletal terms.
A. We agree this is an important alternative explanation. We did not collect a differential leukocyte count or other measure of blood cell composition in this study, and we have added this as an explicit limitation in the Discussion, noting that some DEGs, particularly the structural/cytoskeletal terms in healthy chicks, may reflect shifts in circulating cell-type proportions.

The following sentence was included in the limitation paragraph:
"As in the present study we did not collect a differential leukocyte count or other measure of blood cell composition, some DEGs, particularly the structural/cytoskeletal terms in healthy chicks, may reflect shifts in circulating cell-type proportions and such results should be interpreted with caution."

8.Do the data support "enhance antiviral immunity" and the resistance claim? The central causal claim — that resveratrol enhances antiviral immunity — is inferred entirely from the expression of interferon-stimulated genes; viral load / herpesvirus presence was not measured in the sequenced birds. Elevated ISG expression is equally consistent with resveratrol-treated sick chicks simply carrying a different viral burden, or with among-individual variation in infection stage, especially at these sample sizes. The prediction that responses would be "more pronounced in clinically affected chicks" is also partly confounded with infection state itself (sick chicks have active virus to respond to, irrespective of treatment). I would encourage the authors to (i) temper the title and abstract from a demonstrated increase in "antiviral immunity" toward the transcriptional signature that was actually observed, and (ii) explicitly acknowledge the absence of a viral-load measurement as a limitation for the resistance vs tolerance distinction, which currently reads as more firmly resolved than the data allow.
A. We agree that our data show a transcriptional signature consistent with antiviral pathway activation, but do not directly demonstrate reduced viral load or replication, since viral burden was not measured in the sequenced birds. We have revised the title, abstract, and relevant Discussion statements to describe the observed transcriptional signature rather than a demonstrated increase in antiviral immunity or resistance, and added an explicit statement noting the absence of a viral-load measurement as a constraint on distinguishing resistance from tolerance.

The following paragraph was included in the main text:
Title replaced with:
"Experimental evidence that dietary antioxidants induce an antiviral transcriptomic signature in a wild seabird"

Abstract replaced the last sentence "Collectively, our results elucidate the molecular mechanisms through which dietary antioxidants like resveratrol can act as potent molecular modulators rather than simple passive antioxidants, actively priming host defenses and enhancing cellular resilience. " with:

© 2026. Published by The Company of Biologists under the terms of the Creative Commons Attribution License (https://creativecommons.org/licenses/by/4.0/).

"Collectively, our results reveal a transcriptional signature consistent with dietary antioxidants such as resveratrol acting as active molecular modulators of host defence, rather than simple passive antioxidants, although direct evidence of enhanced antiviral protection (e.g. reduced viral load) remains to be established."

Introduction: rephased the sentence about the prediction into *"We further predicted that these transcriptomic responses would be more pronounced in clinically affected chicks than in apparently healthy chicks, because active disease may increase both immune activation and oxidative stress, thereby increasing the scope for resveratrol to modulate these pathways. Because clinical status itself is expected to influence gene expression, comparisons between health-status groups were interpreted separately from treatment effects."*

Discussion, sentence "The strong upregulation of interferon-stimulated and antiviral genes in clinically affected chicks receiving resveratrol, particularly IFI6, OASL2, and SOCS1, suggests that resveratrol enhances resistance mechanisms by promoting antiviral pathways central to early immune defence." replaced with
"The strong upregulation of interferon-stimulated and antiviral genes in clinically affected chicks receiving resveratrol, particularly IFI6, OASL2, and SOCS1, is consistent with activation of antiviral signalling pathways central to early immune defence, although in the absence of a direct measure of viral load in these birds, we cannot confirm whether this transcriptional response translated into reduced pathogen burden."

Discussion, inclusion of sentence in the limitation paragraph:
"In addition, viral load and herpesvirus presence were not quantified in the sequenced birds; elevated expression of interferon-stimulated genes is consistent with, but does not on its own demonstrate reduced pathogen burden, and it may also partly reflect among-individual variation in infection stage. This constrains our ability to firmly distinguish resistance from tolerance mechanisms in the resveratrol-supplemented sick chicks."

9.Under-use of the group's own oxidative-stress framework. The mechanistic bridge from "antioxidant intake" to "immune gene expression" is left almost entirely to inference from gene names, with CYGB1 (3-fold) as the only directly cytoprotective/anti-oxidative hit. The authors previously measured circulating antioxidant defences, haptoglobin and lipid oxidative damage in this very experiment (Sebastiano et al. 2018), and state in the Methods that corticosterone, oxidative-stress and immune/inflammatory parameters were measured on the D24 samples. Integrating even the already-published oxidative markers with the transcriptomic groups (e.g. relating individual antioxidant/oxidative-damage values to the ISG response) would materially strengthen the antioxidant→immunity argument and is more in keeping with the mechanistic claim in the title. As it stands, the oxidative-stress mechanism is asserted rather than shown.
A. We agree that connecting the transcriptomic signal to our previously published oxidative stress and antioxidant defence data (Sebastiano et al. 2018) strengthens the mechanistic narrative, and we have expanded the Discussion to explicitly link the D24 antioxidant/oxidative damage findings from that study to the immune gene expression patterns reported here. However, we consider a full individual-level statistical integration of the two datasets to be beyond the scope of the present transcriptomic study. But we will state how this integrative analysis represents an important direction for future work.

The following paragraph was included in the main text:
"In that earlier study using the same experimental population, resveratrol-supplemented chicks showed higher circulating antioxidant defences and plasma haptoglobin, and reduced lipid oxidative damage, relative to placebo-treated birds (Sebastiano et al., 2018). The present transcriptomic results extend those physiological findings to the molecular level: the upregulation of CYGB1, a cytoprotective gene induced by oxidative and hypoxic stress, together with the broader antiviral and immunoregulatory signature described above, is consistent with a mechanistic link between antioxidant intake and reduced immune-associated cellular damage. We did not, however, test this link statistically at the individual level (e.g. by relating antioxidant or oxidative-damage values to gene expression for each bird), and consider such an integrated analysis an important next step rather than a claim supported by the present dataset."

© 2026. Published by The Company of Biologists under the terms of the Creative Commons Attribution License (https://creativecommons.org/licenses/by/4.0/).

STATISTICS
*DESeq2 design formula. The Methods do not give the design formula. Were any covariates included (sex, chick age/beak-head length, RNA quality/RIN, sequencing batch)? Sex in particular is worth checking, as it commonly structures blood transcriptomes; if it could not be modelled given n, please say so.
A. We thank the reviewer for raising this. The DESeq2 design formula used the factor condition, comparing resveratrol-treated and placebo (control) chicks within each health-status group (sick, healthy, pooled). No additional covariates (sex, age/morphometrics, RNA integrity, sequencing batch) were included in the model. Sex was not recorded for the sequenced birds as at this age they do not differ in physiology or morphology. Given group sizes as low as three per group, we agree that adding further covariates was in any case not statistically feasible, and we have stated this explicitly in the Methods (see below).

The following paragraph was included in the methods:
Differential expression was assessed using DESeq2 (1.42.0) with a simple one-factor design (~condition), comparing resveratrol-supplemented and placebo-treated chicks separately within sick, healthy, and pooled groups. No additional covariates were modeled. Sex was not included because it was not determined for the sequenced individuals.

*"Fold change above 1" wording. The threshold is stated as "adjusted p-values <0.05 and absolute fold change above 1 (or below -1) - corresponding to at least a twofold difference". A fold change of 1 is no change; you mean |log2 fold change| > 1. Please correct the wording to log2FC to match Table 1's columns.
A. Thanks for spotting the mistake. The sentence was corrected.

*Multiple contrasts. Three separate DESeq2 contrasts (sick, healthy, pooled) are run and interpreted. The pooled result largely recapitulates the sick result (as the authors note, sick chicks are >50% of the pool); it would help to state clearly that the pooled contrast is not independent evidence.
A. We agree and have added a sentence noting that the pooled contrast is not statistically independent of the sick-chick contrast, since sick chicks make up >50% of the pooled sample.

*Report the number of genes tested and the independent-filtering outcome for each contrast, so the reader can judge the multiple-testing burden behind the padj values.

MINOR COMMENTS

*Table 1: the two "NA"/novel transcripts (FREMAG_R14896, FREMAG_R14315) are counted within the "10 DEGs" in sick chicks but excluded from interpretation. Please state the DEG counts consistently (e.g. "10 DEGs, of which 2 are unannotated novel transcripts") wherever the number is used.
A. The information has been included as suggested.

*Table 1 / text: DDX60 and CYGB1 are described as "upregulated by 7 and 3 folds"; please make sure the fold values quoted in the text match the Fold-change column of Table 1 throughout, and give the direction consistently.
A. We double checked but everything was correct.

*Gene-name spelling: "OASL2 (2'-5'-Oligoadenilato Sintetasi 2)" uses the Italian spelling; the standard English is "2'-5'-oligoadenylate synthetase-like 2". Please standardise gene descriptions to the official symbols/names (e.g. HGNC), and clarify the "_0"/"_1" suffixes (point 4-ii).
A. The gene name was corrected and the suffixes clarified in the main text.

*Citation for the reference genome: replace "(Mortazavi et al., 2008)" for genome alignment with the correct genome assembly reference (point 4-i). Also double-check that the intended aligner citation (HISAT2, Kim et al.) is the one attached to "Hisat2 v2.0.5".
A. The relevant reference (Kim et al. 2019) was included in the text.

*Methods, sample selection: the RNA-seq subset is "20 birds with clear health history" drawn from 60. Please state the selection criteria and confirm these 20 are representative (e.g. not selected on disease severity or RNA yield), as non-random selection would bias the treatment contrasts.

© 2026. Published by The Company of Biologists under the terms of the Creative Commons Attribution License (https://creativecommons.org/licenses/by/4.0/).

A. We have now included additional information.

*Please report RNA integrity (RIN) values per sample (e.g. in the supplement), and confirm whether any samples were excluded on quality grounds. Read depth per sample and mapping rates would also be useful for assessing rigour.
A. We thank the reviewer. RNA integrity (RIN) values for all 22 samples that underwent RNA extraction and QC are now reported in Supplementary Table S1. All samples passed Novogene's standard QC criteria (RIN range: 4.0–7.2).

*Methods say plasma was used for corticosterone, oxidative-stress and immune/inflammatory parameters; since none are reported here, either integrate them (point 9) or cross-reference where they are/will be reported, so the reader is not left expecting them.
A. The relevant reference has been included in the manuscript.

*"CYGB1" — cytoglobin is normally CYGB; please confirm the "1" is a genuine paralog and not an annotation suffix, since it is used as a substantive cytoprotection argument.
A. Although humans only possess one CYGB, in other vertebrates the presence of distinct copies of this gene has been confirmed so it is a paralog as explained in details above.

*Consider stating the herpesvirus context more carefully: the disease is described as herpesvirus-associated (de Thoisy et al. 2009) but not confirmed in the study birds; wording such as "likely actively replicating" should be flagged as an assumption.
A. The sentence was rephrased and toned down.

Reviewer 2

Reviewer 2: This is an interesting study examining the impact of resveratrol supplementation on gene expression changes in healthy and sick Magnificent frigatebirds, and its potential impact on immune health in this species.
A. We thank the reviewer for the positive evaluation of our work and the constructive comments provided, which helped to improve the quality and clarity of the manuscript.

2.Reproducibility
a.Were experiments in each figure performed using adequate number of biological replicates?
Yes.
b.Is there sufficient raw data to assess the rigor of the analysis?
No.
It is standard practice to deposit raw transcriptome sequencing data in a public sequencing repository, for example, the NCBI Sequence Read Archive under a BioProject accession number. Then interested readers can analyse the data themselves.
A. We thank the reviewer for raising this important point. The raw sequencing reads for all 20 samples have been deposited in the NCBI Sequence Read Archive under BioProject accession PRJNA1519313. The gene-level count matrices are provided as a supplementary Excel table , and the DESeq2 analysis workflow (including the design formula, filtering, and shrinkage procedure) is fully described in the Methods, allowing readers to inspect the count distributions underlying the reported fold changes. We have added a Data Availability statement to the manuscript.

There has been an attempt to include the results of the DEG analysis on PDF pages 19-66. I think this includes the background gene set used for KEGG enrichment; however, it is extremely difficult to interpret. It appears as though Excel data has been pasted into the Word format, so you have connected information spanning several pages of the manuscript. It would be far better to provide this information as a supplementary table in Excel.
A. We agree and we have also replaced the pasted DEG/KEGG background gene list (previously spanning PDF pages 19–66 due to an upload error) with a proper Supplementary Excel table.

c.Does the methods section provide sufficient detail to permit reproducibility?
Not fully. Methods requiring more details are:
No details are given on the blood processing and RNA isolation method (line 155).

© 2026. Published by The Company of Biologists under the terms of the Creative Commons Attribution License (https://creativecommons.org/licenses/by/4.0/).

The methods jump from library preparation (lines 158-160) straight to sequence read filtering/mapping/analysis. There is no mention of how the sequencing was done. Please add this information.
A. We agree these details were missing and have now included them in the specific section as requested.

Line 160: "real-time PCR for quantification and bioanalyzer". Neither of these methods have sufficient detail provided to permit reproducibility. For "bioanalyzer", do you mean an Agilent bioanalyser? So, a different analyser to the 5400 fragment analyser indicated on line 155?
A. This information has been included in the manuscript.

Lines 182-191: In the Methods subsection "Enrichment analyses of differentially expressed genes", please specify all the analysis tools, databases, and software packages used.
A. This information has been included in the manuscript.

3.Completeness
a.Are the author's conclusions supported by the data?
Generally, yes. However, the discussion centred around the specific DEGs identified (lines 297-348) should make clear that this is a relatively small group of genes. Therefore, interpretations should be cautious, and conclusions drawn can only be validated by repeating the experiment alongside further, complementary, experiments. Or words to this effect.
A. We thank the reviewer for the constructive comment. We have now worked on the manuscript to address the limitations of the study also in consideration of the small sample size, and we have also interpreted our results in a more cautious way.

The manuscript title has "enhance antiviral immunity", which feels quite a bold description. I would prefer something a bit more toned down to indicate resveratrol affects the expression of genes with known roles in antiviral immunity.
A. The title has been modified as suggested and it appears now as "Experimental evidence that dietary antioxidants induce an antiviral transcriptomic signature in a wild seabird"

b.Are there any flaws in the experimental design that invalidate the approach taken by the authors?
Nothing obvious to me. The experimental design appears to be robust.
I am interested to know how simple/challenging capturing the birds for repeated sampling is?
A. Thanks for the comment. Repeated sampling of chicks is easily achievable in this species as they do not move from their nests until they are a few months old. This information has been included in the manuscript.

c.Are there experiments that have not been performed, but if true would disprove the conclusion? If yes, and if such experiments would be costly or time-consuming to perform, do the authors acknowledge this in a discussion of the limitations?
Additional experiments that could be performed and are not mentioned in the discussion (they would be costly):
Single-cell transcriptomics on PBMCs to determine which subpopulations exhibit the greatest transcriptional response. This may identify other DEGs masked by the bulk sequencing approach.
Combining transcriptome analysis with proteomics (a good method of validating the biological relevance of the highly expressed genes identified).
A few sentences could be added to acknowledge these.
A. We agree these would be valuable complementary approaches and have added a sentence to the Discussion noting that single-cell transcriptomics and proteomic validation of key transcripts would be valuable next steps, while acknowledging their cost as a reason why they were not undertaken here.

The following sentence was included in the manuscript:
"Future work could extend these findings using single-cell transcriptomics on peripheral blood mononuclear cells to identify which specific cell subpopulations drive the observed transcriptional response, potentially revealing additional differentially expressed genes masked by the bulk sequencing approach used here. Furthermore, combining transcriptomic data with proteomic validation of the key transcripts identified would confirm their

© 2026. Published by The Company of Biologists under the terms of the Creative Commons Attribution License (https://creativecommons.org/licenses/by/4.0/).

biological relevance at the protein level. Both approaches were beyond the scope and resources of the present study."

4.Scholarship
a.Do the authors cite and discuss the merits of relevant data that would argue against their conclusion?
No, however, the points they discuss are relevant and clear and appropriately cited. The authors also included this in the discussion "However, because these patterns emerged only in pooled analyses, future studies with larger sample sizes and longitudinal designs will be necessary to determine whether these transcriptional shifts represent early regulatory responses that precede phenotypic changes in disease progression". Which I feel adequately acknowledges the limitation of the findings.
A. We thank the reviewer for this comment. We have now also better discussed some other limitations of our findings as well as future research directions to be explored.

Specific points that require attention:
Line 157-160 - was a specific kit used for the library prep?
A. Yes, NEBNext Ultra RNA Library Prep Kit for Illumina. This info has been included in the manuscript.

Lines 165-166: "All the downstream analyses were based on the clean data with high quality". The English could be improved here. For example. "All downstream analyses used only high quality clean data".
A. The sentence has been modified as suggested.

Line 202-203: You excluded two (highly expressed) unannotated transcripts. You later (on line 219), mention this was because one of these (FREMAG_R14896) lacked known orthologues. Was this also the case with FREMAG_R14315?
A. Yes. As with FREMAG_R14896, FREMAG_R14315 lacked a known orthologue in the reference annotation used (both are annotated only by their locus identifier, with no gene symbol assigned by the B10K comparative annotation pipeline). We have now clarified this explicitly in the Results, naming both transcripts together where they are first excluded from interpretation, rather than only specifying the reason for one of them.

The sentence in the result section now stands as:

"After excluding two novel, unannotated transcripts (FREMAG_R14896 and FREMAG_R14315, both of which lack known orthologues in the reference annotation and were therefore not considered for biological interpretation; Table 1), the IFI6 (Interferon alpha Inducible Protein 6) gene showed the greatest increase in expression..."
Line 343: please remove the word "significant".
A. The word has been removed as suggested.

When describing fold changes in expression, the plural form of the word "folds" is used repeatedly. The singular form "fold" is the correct one to use. Please amend.
A. We applied the corrections as suggested.

Was ethical approval required for this study? If so, details are missing. Please specify or clearly indicate which reference has this information.
A. Yes. The following sentence has been included in the manuscript:
"All procedures were approved by the Ethical Committee of the University of Antwerp and by DEAL Guyane (Direction de l'Environnement, de l'Aménagement et du Logement), and fieldwork was conducted under permit R03-2013-06-01-007 issued by the Préfet de la Région Guyane."

Did the authors consider carrying out something like weighted gene co-expression network analysis (WGCNA)? Or a different type of gene regulatory network analysis? Or would it not have added much and been largely speculative with this data set?

© 2026. Published by The Company of Biologists under the terms of the Creative Commons Attribution License (https://creativecommons.org/licenses/by/4.0/).

A. We considered WGCNA-type network analyses but concluded that with total sample sizes of 20, a co-expression network approach would be statistically underpowered and largely speculative; we therefore did not pursue this and have noted it as a possible direction for a larger future dataset.

Second decision letter

MS ID#: bio.062786R1

MS Title: Experimental evidence that dietary antioxidants induce an antiviral transcriptomic signature in a wild seabird

Authors: Manrico Sebastiano; Marcel Eens; Olivier Chastel; Louis Maigné; David Costantini

I have now reached a decision on the above manuscript.

The reviewer reports are shown at the bottom of this email.

As you will see, the reviewers gave favourable reports, but raised a few points that will require amendments to your manuscript. I hope that you will be able to carry these out, because we would like to be able to accept your paper.

At this stage, we also ask you to ensure your manuscript complies with our formatting guidelines – please see our manuscript preparation guidelines for details. Provided you are able to fully address the referees' comments, we are positive about publication of your paper (we accept over 95% of revision submissions) and therefore hope you won't mind any extra work involved in reformatting your manuscript at this point.

Please upload both a 'clean' version of your Word file, along with a highlighted version clearly showing where you have made changes in the revised manuscript. Please avoid using 'Track changes' in Word files as these are lost in PDF conversion.

I should be grateful if you would also provide a point-by-point response detailing how you have dealt with the points raised by the reviewers in the 'Response to Reviewers' box. Please attend to all of the reviewers' comments. If you do not agree with any of their criticisms or suggestions please explain clearly why this is so.

Reviewer 1

Comments for the author

I reviewed the original submission of this manuscript and raised several concerns that have been properly addressed so I think it is close to being acceptable. I have a small number of remaining points:

The Results text still quotes the raw fold changes, which no longer match Table 1. This comes directly from the switch to apeglm effect sizes.

The methods state that "shrunken values are reported throughout in place of raw maximum-likelihood estimates," and Table 1 has been updated accordingly but the Results text still needs fixing.

The Results state "we found a total of 3 differentially expressed genes (Table 1...)" for the healthy contrast, but Table 1 lists only ENTPD3 and SPTBN4.

© 2026. Published by The Company of Biologists under the terms of the Creative Commons Attribution License (https://creativecommons.org/licenses/by/4.0/).

I understand that the apeglm shrinkage collapsed its log2 fold change to approximately zero and this exclusion is explained in the methods but not in the Results, so the "3 DEGs" statement is not right.

Could you add a version number or date accessed to the AI disclosure part so readers would know witch version was used.

Reviewer's Responses to Questions

Experimental quality
Are experiments performed using appropriate methods that will answer the question (or test the hypothesis or support the observations) posed by the authors? Is the right tool used for the job?
If 'No', please indicate reasons in Comments for Author box below.
Reviewer #1:

- Yes

Were the data analyzed using appropriate statistical tests?
If 'No', please indicate reasons in Comments for Author box below.
Reviewer #1:

- Yes

Reproducibility
Were experiments performed using adequate number of biological replicates?
If 'No', please indicate reasons in Comments for Author box below.
Reviewer #1:

- Yes

Is there sufficient raw data to assess the rigor of the analysis?
If 'No', please indicate reasons in Comments for Author box below.
Reviewer #1:

- Yes

Does the methods section provide sufficient detail to permit reproducibility?
If 'No', please indicate reasons in Comments for Author box below.
Reviewer #1:

- Yes

Completeness
Are the manuscript's conclusions supported by the data?
If 'No', please indicate reasons in Comments for Author box below.
Reviewer #1:

- Yes

Scholarship
Do the authors cite and discuss the merits of data that would argue for and against their conclusion?
If 'No', please indicate reasons in Comments for Author box below.
Reviewer #1:

- Yes

© 2026. Published by The Company of Biologists under the terms of the Creative Commons Attribution License (https://creativecommons.org/licenses/by/4.0/).

Does the manuscript title & abstract accurately reflect the contents of the manuscript, without hyperbole?
If 'No', please indicate reasons in Comments for Author box below.
Reviewer #1:
- Yes

Second revision

Author response to reviewers' comments

Submission of manuscript ID bio.062786R1 after revision

Dear Editor,

Please, find enclosed a revised version of our manuscript entitled "Experimental evidence that dietary antioxidants induce an antiviral transcriptomic signature in a wild seabird" by myself, Marcel Eens, Olivier Chastel, Louis Maigné, and David Costantini, submitted for reconsideration for publication in Biology Open.

We would like to thank you and the reviewers for the time and effort devoted to evaluating our manuscript and for the positive evaluation of our work. Below, we provide a detailed, point-by-point response to each of the remaining minor remarks from the reviewers. Our responses are indicated in bold and preceded by the abbreviation "A".

We hope that the revised version will now be suitable for publication in Biology Open and will be of interest to the journal's readership.

Reviewer 1

Reviewer 1: I reviewed the original submission of this manuscript and raised several concerns that have been properly addressed so I think it is close to being acceptable. I have a small number of remaining points:

A. We thank the reviewer for the positive evaluation of our work. We have now addressed the final comments and changed the manuscript accordingly.

The Results text still quotes the raw fold changes, which no longer match Table 1. This comes directly from the switch to apeglm effect sizes.
The methods state that "shrunken values are reported throughout in place of raw maximum-likelihood estimates," and Table 1 has been updated accordingly but the Results text still needs fixing.
A. We thank the reviewer for noticing this mismatch. We have now updated the text accordingly.

The Results state "we found a total of 3 differentially expressed genes (Table 1...)" for the healthy contrast, but Table 1 lists only ENTPD3 and SPTBN4.
I understand that the apeglm shrinkage collapsed its log2 fold change to approximately zero and this exclusion is explained in the methods but not in the Results, so the "3 DEGs" statement is not right.
A. We thank the reviewer for pointing that out. We have now corrected the sentence accordingly.

Could you add a version number or date accessed to the AI disclosure part so readers would know which version was used.
A. The information has been included as requested.

© 2026. Published by The Company of Biologists under the terms of the Creative Commons Attribution License (https://creativecommons.org/licenses/by/4.0/).

Third decision letter

MS ID#: bio.062786R2

MS Title: Experimental evidence that dietary antioxidants induce an antiviral transcriptomic signature in a wild seabird

Authors: Manrico Sebastiano; Marcel Eens; Olivier Chastel; Louis Maigné; David Costantini

I am happy to tell you that your manuscript has been accepted for publication in Biology Open, pending our standard publication integrity checks. It was accepted on 21st September 2026.

© 2026. Published by The Company of Biologists under the terms of the Creative Commons Attribution License (https://creativecommons.org/licenses/by/4.0/).
